# Lineage-specific roles of the cytoplasmic polyadenylation factor CPEB4 in the regulation of melanoma drivers

**DOI:** 10.1038/ncomms13418

**Published:** 2016-11-18

**Authors:** Eva Pérez-Guijarro, Panagiotis Karras, Metehan Cifdaloz, Raúl Martínez-Herranz, Estela Cañón, Osvaldo Graña, Celia Horcajada-Reales, Direna Alonso-Curbelo, Tonantzin G. Calvo, Gonzalo Gómez-López, Nicolas Bellora, Erica Riveiro-Falkenbach, Pablo L. Ortiz-Romero, José L. Rodríguez-Peralto, Lorena Maestre, Giovanna Roncador, Juan C. de Agustín Asensio, Colin R. Goding, Eduardo Eyras, Diego Megías, Raúl Méndez, María S. Soengas

**Affiliations:** 1 Melanoma Group, Molecular Oncology Programme, Spanish National Cancer Research Centre (CNIO), Madrid 28029, Spain; 2 Bioinformatics Unit (CNIO), Madrid 28029, Spain; 3 Department of Dermatology, Hospital Gregorio Marañón, Madrid 28003, Spain; 4 Department of Experimental and Health Sciences, Universidad Pompeu Fabra, Barcelona 08002, Spain; 5 Instituto de Investigación i+12, Hospital 12 de Octubre, Medical School, Universidad Complutense, Madrid 28041, Spain; 6 Monoclonal Antibodies Unit, Biotechnology Programme (CNIO), Madrid 28029, Spain; 7 Department of Pediatric Surgery, Hospital Gregorio Marañón, Madrid 28003, Spain; 8 Institute for Cancer Research, Nuffield Department of Medicine, University of Oxford, Oxford OX3 7DQ, Spain; 9 Institució Catalana de Recerca i Estudis Avançats (ICREA), Barcelona 08010, Spain; 10 Confocal Microscopy Unit, (CNIO) Madrid 28029, Spain; 11 Translational Control of Cell Cycle and Differentiation Group, Molecular Medicine Department, Institute for Research in Biomedicine (IRB), The Barcelona Institute of Science and Technology, Barcelona 08028, Spain

## Abstract

Nuclear 3'-end-polyadenylation is essential for the transport, stability and translation of virtually all eukaryotic mRNAs. Poly(A) tail extension can also occur in the cytoplasm, but the transcripts involved are incompletely understood, particularly in cancer. Here we identify a lineage-specific requirement of the cytoplasmic polyadenylation binding protein 4 (CPEB4) in malignant melanoma. CPEB4 is upregulated early in melanoma progression, as defined by computational and histological analyses. Melanoma cells are distinct from other tumour cell types in their dependency on CPEB4, not only to prevent mitotic aberrations, but to progress through G1/S cell cycle checkpoints. RNA immunoprecipitation, sequencing of bound transcripts and poly(A) length tests link the melanoma-specific functions of CPEB4 to signalling hubs specifically enriched in this disease. Essential in these CPEB4-controlled networks are the melanoma drivers MITF and RAB7A, a feature validated in clinical biopsies. These results provide new mechanistic links between cytoplasmic polyadenylation and lineage specification in melanoma.

## Abstract (web summary)

Cytoplasmic polyadenylated transcripts have been poorly characterized, particularly in cancer. Here the authors identify a lineage-specific requirement of the cytoplasmic polyadenylation binding protein 4 (CPEB4) in malignant melanoma and show that it controls melanoma drivers MITF and RAB27A.

## Graphical abstract

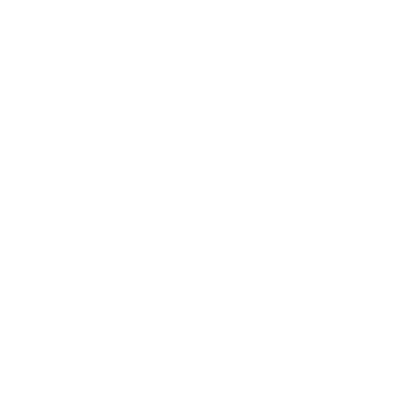

In the nucleus, nearly all nascent mRNAs become polyadenylated at their 3′-end (ref. 1). In the cytoplasm, shortening of the poly(A) tail usually results in cessation of translation, decapping and mRNA destabilization^2^. However, a subset of mature mRNAs can sustain short poly(A) tails, remaining stable and translationally silent until reactivated by re-adenylation^2^,^3^. While the identity of the transcripts regulated by cytosolic polyadenylation and the macromolecular complexes involved are still not completely defined, main effectors are the four members of the cytoplasmic polyadenylation element binding family (that is, CPEB1 to CPEB4). Initially described as essential modulators of meiosis in *Xenopus laevis*, CPEBs have been also reported with key roles in somatic cells, for example in the control of mitosis, differentiation, cell polarity or motility, among others^3^,^4^,^5^. These processes can favour cancer, and therefore CPEBs are raising great interest in oncology for their potential impact on post-transcriptional mechanisms of gene reprogramming associated with tumour development^4^,^5^,^6^,^7^. Nevertheless, the assignment of specific functions to individual CPEBs has been challenging. Thus, RNA-binding domains of CPEBs are highly conserved, with the potential for overlap in transcript recognition^8^,^9^. However, differential roles of CPEBs in developmental processes suggest that in mammalian systems, CPEB substitution/compensation is not a generalized mechanism^10^,^11^,^12^,^13^, although the specific identity of the targets involved remains unclear. Moreover, the expression of CPEBs in cancer cells is highly variable, with pro- and anti-tumorigenic roles reported depending on the system^4^. For example, suppressive functions of CPEB1 in fibroblasts^14^ or CPEB4 in hepatic cancer cells^15^ contrast with cooperating effects of these proteins to favour cell cycle progression in HeLa or 293 cells^16^,^17^. The underlying basis for these seemingly opposing roles are unclear, but may reflect the fact that CPEBs can modulate both 3′-end mRNA adenylation and deadenylation^4^.

A disease where the functional impact of the CPEB-mediated translational programming remains unexplored is malignant melanoma. Melanomas are a paradigm of aggressive tumours characterized by the largest mutational rate described to date^18^, and the accumulation of massive changes in the transcriptome^19^. Still, it is unknown whether these alterations affect or are modulated by cytoplasmic polyadenylation. Similarly, putative functions of poly(A) tail length alterations remain unexplored in the context of lineage-specification. This is relevant as melanoma is a prototype of disease, where tumour cells rewire intrinsic developmental traits to favour proliferative and metastatic capabilities^20^,^21^,^22^. In this context, the best characterized melanocytic-specific oncogene is the microphthalmia-associated transcription factor (MITF)^23^,^24^,^25^. Intriguingly, MITF can be subject to highly dynamic regulatory mechanisms that either activate or inhibit expression and function depending on the specific stage of tumour progression^26^,^27^,^28^,^29^. Whether MITF mRNA undergoes cytoplasmic polyadenylation is unknown. This lack of information also applies to other lineage-specific pathways driven by vesicular trafficking modulators such as RAB proteins, recently emerging as key components of gene clusters selectively enriched and required by melanoma cells^30^,^31^,^32^,^33^.

To date, the only information on CPEB proteins in melanoma is limited to CPEB1 as the target of the tumour suppressor miR-455-5p (ref. 34). Nevertheless, cellular factors affected by CPEB1-controlled cytoplasmic polyadenylation have yet to be defined. Similarly, there are no reports of the CPEB family members CPEB2, CPEB3 and CPEB4 in melanoma. Nevertheless, CPEB4 is particularly attractive for its overexpression in gliomas and pancreatic cancer^35^. In these tumours, CPEB4 was not strictly necessary for cell proliferation, but was found to act in a non-cell autonomous manner remodelling the stroma and favouring angiogenesis in mouse xenografts^35^. However, as mentioned above, these roles of CPEB4 (and the corresponding transcripts involved) may not be universal, in the light of the downregulation of this protein in hepatocellular carcinomas^15^, gastric cancer or haematological diseases^4^.

Performing a computational analysis of CPEB proteins across tumour types, we found a high expression of CPEB4 in melanoma cells. Histological and functional analyses demonstrated a cell autonomous and lineage-specific dependency of melanomas on CPEB4, in a manner not shared by other tumour cell types. These data identify novel melanoma-associated vulnerabilities in this otherwise histopathologically complex disease.

## Results

### CPEB4 is upregulated early during melanoma development

**Figure 1:**
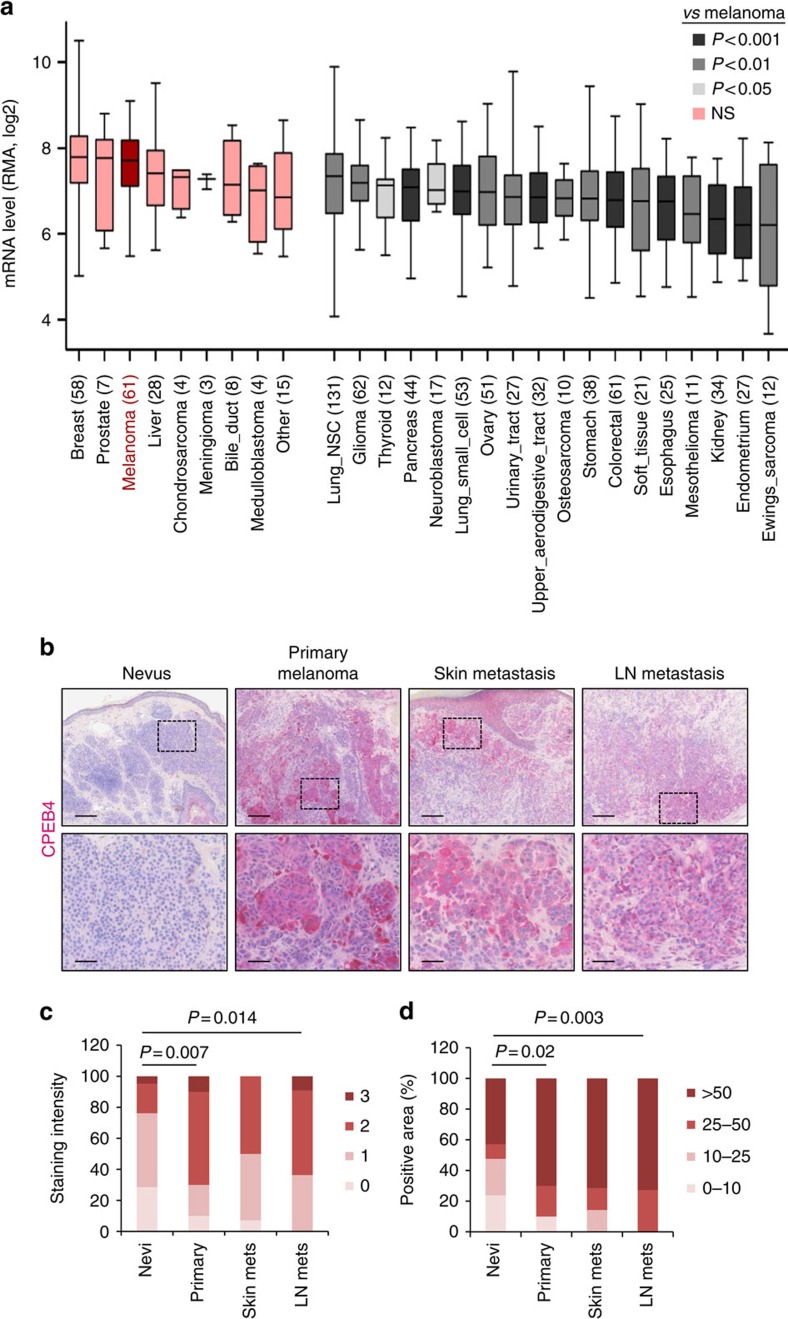
CPEB4 is induced at early stages of melanoma development. (**a**) *CPEB4* mRNA expression in 27 solid tumours including melanoma extracted from the CCLE data set (CCLE_Expression_Entrez_2012-10-18.res). mRNA levels are normalized by inter-sample variability across the cell lines included in the data set (RMA-Log2). The number of cell lines from each cancer type is indicated in parenthesis. Box colours represent the *P-*values from pairwise comparisons between melanoma and each tumour type. (**b**) Representative micrographs of sections from the indicated lesions showing CPEB4 staining in pink. Nuclei are counterstained with hematoxylin. Scale bars, 200 μm (upper images); and 50 μm (lower images). (**c**) Quantification of CPEB4 staining intensity in the indicated lesion type scored from 0 (negative) to 3 (highest). The number of lesions per category is indicated in Supplementary Methods. (**d**) Percentage of CPEB4-positive cells in melanoma lesions (four independent areas were analysed by stained specimen, using hematoxylin as a reference to estimate total cell number per section; epidermal and stromal cells were excluded from the analysis). χ^2^-test *P* values (*P*) are indicated. NS, non-significant.

One of the defining features of melanomas is their inherent metastatic potential^36^. Pro-angiogenic roles of CPEB4 identified in pancreatic adenocarcinomas and other pathologies^11^,^35^ were of interest as possible contributors to the aggressive behaviour of advanced melanomas. CPEB4 was also attractive as in cancer, this is the only CPEB for which antibodies have been validated for genome-wide RNA immunoprecipitation and target identification^35^. Publicly accessible transcriptomic profiles were mined for a global evaluation of *CPEB4* mRNA expression in melanoma and to identify possible differences with other malignancies. The Cancer Cell Line Encyclopedia (CCLE) offered an ideal platform for comparative analyses as it covers over 1,000 cell lines of 37 different cancer types collected and processed in a uniform manner^37^. Focusing on solid tumours, melanoma cells were found among the highest expressors of *CPEB4* (Fig. 1a). Intriguingly, while CPEB4 was not exclusive of melanoma, pairwise analyses identified statistically significant differences with 18 tumour types, including glioblastoma and pancreatic cancer (Fig. 1a), where CPEB4 functions and targets have been addressed in more detail. High *CPEB4* mRNA in melanoma cells was supported by two independent data sets^38^,^39^ corresponding to human clinical biopsies (*N*=198 and 119 cases, respectively) that span across various cancer types (see Supplementary Fig. 1a). These results were further validated by quantitative PCR after reverse transcription (qRT-PCR) in a panel of cell lines from melanoma and other tumour types (Supplementary Fig. 1b). Histological analyses were then performed on tissue microarrays containing representative examples of 15 tumour types (*N*=108 specimens; see Supplementary Information for detail). This analysis confirmed a high CPEB4 expression in melanoma, although again, not restricted to this disease (see Supplementary Fig. 1c for comparative analyses with other malignancies with strong, medium and low CPEB4 staining).

Next, biopsies from lesions containing non-malignant melanocytes (nevi) and from melanomas at different stages of progression (Fig. 1b; *N*=56), were analysed to define whether CPEB4 upregulation is an early or late event in this disease. Given the roles of CPEB4 in the control of pro-metastatic factors such as tissue plasminogen activator^35^ and the vascular epithelial growth factor^11^, we had anticipated an increased expression of CPEB4 towards advanced melanomas. However, CPEB4 was found upregulated already at early stages in melanoma development (Fig. 1c,d). Therefore, we questioned whether this early induction of CPEB4 reflects in the control of essential genes for cell proliferation, and to which extent these functions are shared with other tumour types.

### Non-redundant roles of CPEB4 in melanoma cell proliferation

**Figure 2:**
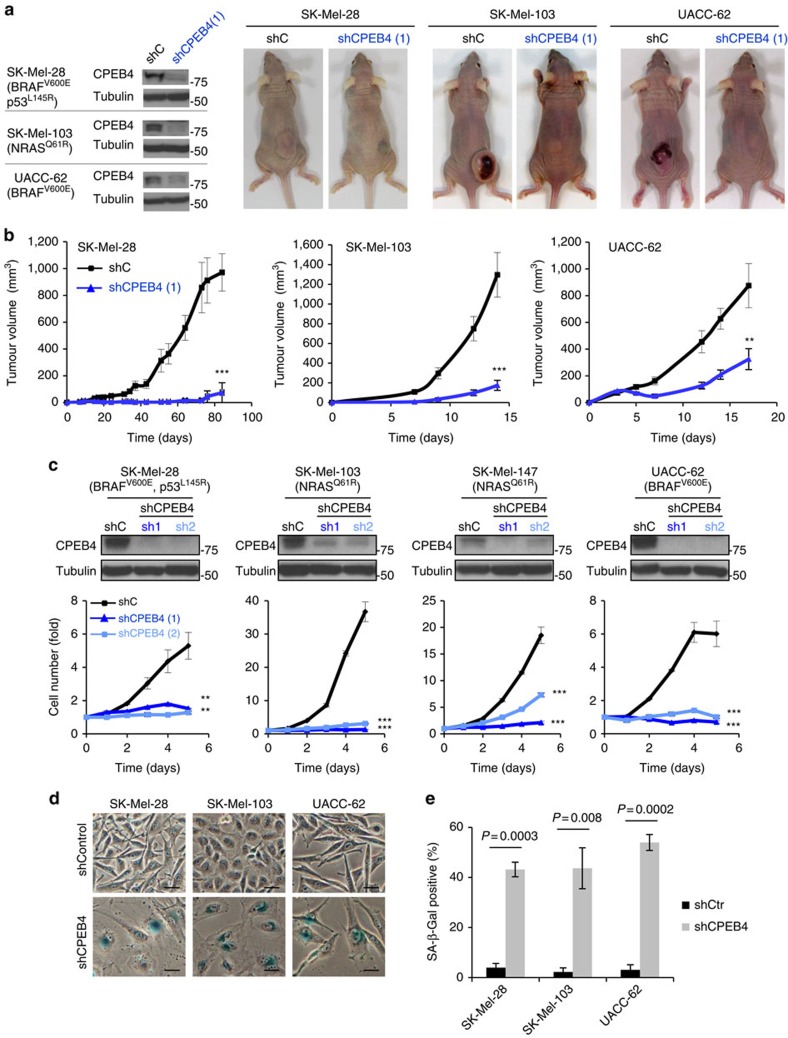
Essential cell-autonomous role of CPEB4 in melanoma growth. (**a**) Representative examples of animals subcutaneously implanted with SK-Mel-28, SK-Mel-103 or UACC-62 cells expressing control shRNA (shC) or CPEB4 shRNA (sh1). Key melanoma-associated mutations in the indicated cell lines are listed in parenthesis. Panels on the left correspond to immunoblots for visualization of the efficiency of the CPEB4 shRNA-depleting constructs in the different cell lines. (**b**) Differential tumour growth upon subcutaneous implantation of cells in immunosuppressed mice in **a**. *N*=5 mice per group. (**c**, upper) Depletion of CPEB4 in the indicated cell lines upon lentiviral-driven transduction of two validated shRNA for CPEB4 (sh1 and sh2) shown by immunoblotting using cells expressing a shRNA control (shC) as a reference. (lower) Melanoma cell proliferation after transduction of control or CPEB4 shRNAs. Graphs depict relative cell numbers at the indicated time points obtained from three independent experiments in triplicate. (**d**) Representative images of SA-β-Gal staining (blue) of the indicated melanoma cell lines expressing control or CPEB4 shRNAs. Scale bars, 20 μm. (**e**) Percentage of SA-β-Gal-positive cells from **d**. Error bars represent s.e.m. from three independent experiments. Student's *t*-test and ANOVA *P* values are indicated or represented as ***P<0.001, **P<0.01. ANOVA, analysis of variance.

Next, we took advantage of two previously validated short hairpin RNAs (shRNAs)^35^ to deplete CPEB4 in a panel of melanoma cell lines that recapitulate frequent characteristic oncogenic mutations in BRAF, NRAS or p53 of this disease (see depletion efficacies in Fig. 2a). Control and shRNA-expressing cells were injected subcutaneously in immunosuppressed mice. Curiously, 90% of implants of shCPEB4-cells failed to grow (Fig. 2a,b), halting progression even before sizable lesions could be generated that needed additional roles on vascularization. This was unexpected considering the reported situation in pancreatic cancer cells, where CPEB4 depletion delayed, but did not abrogate xenograft formation^35^.

**Figure 3:**
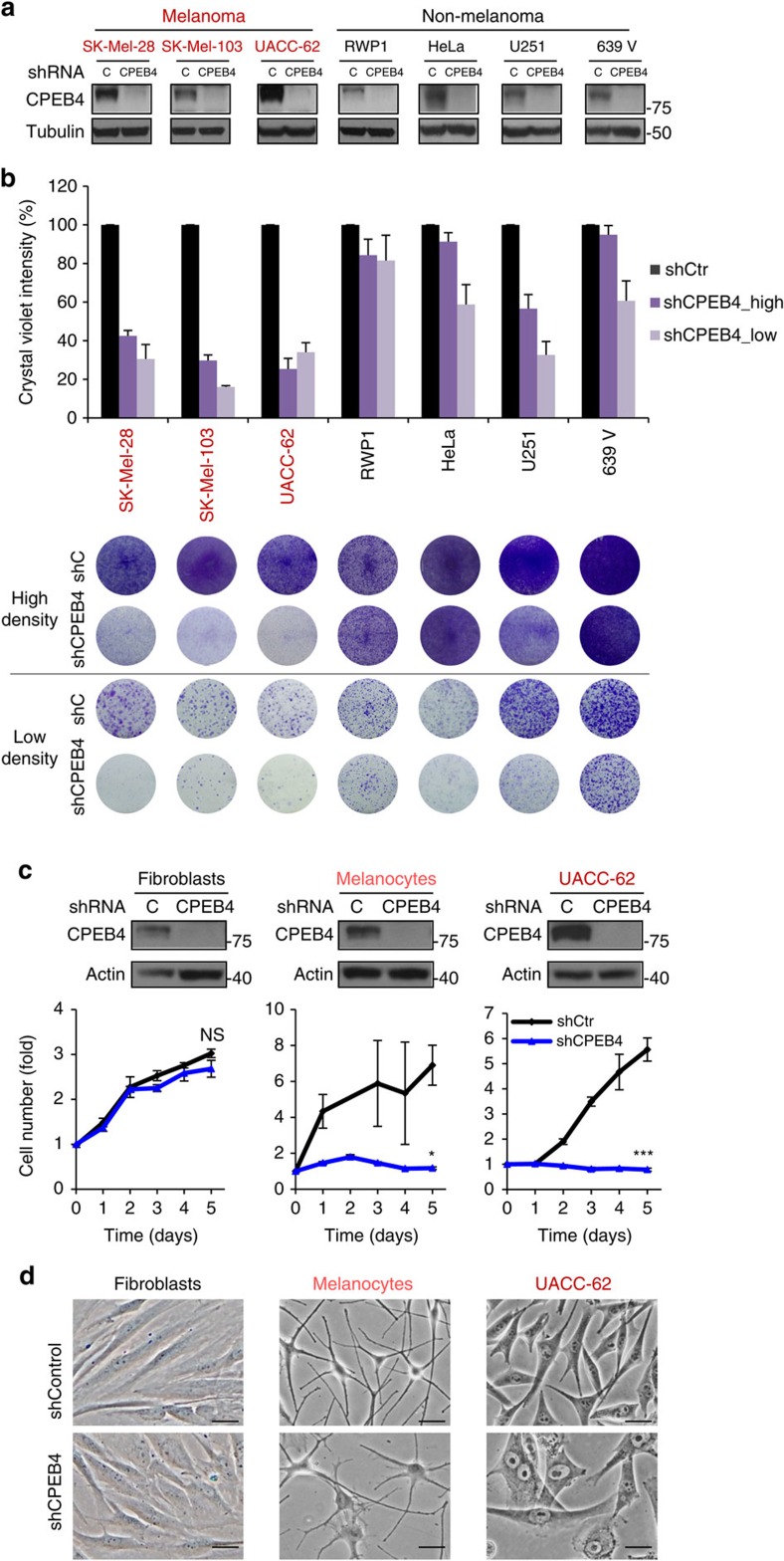
Increased sensitivity of melanoma cells to CPEB4 depletion. (**a**) Immunoblots showing CPEB4 levels upon transduction of control or CPEB4 shRNAs in a panel of melanoma (red) and non-melanoma (black) cell lines. (**b**) Colony formation assays of the indicated tumour cell lines seeded at high or low density (5 × 10^4^ or 2–4 × 10^3^ cells, respectively). Bar graphs correspond to cell number estimated by crystal violet staining and represented with respect to shC-transfected cells. Data are plotted as means±s.e.m. of three independent experiments in duplicate. (**c**) Depletion of CPEB4 (by shRNA) in genetically matched pairs of human skin fibroblasts and melanocytes visualized by immunoblotting (upper) with respect to shC-transduced cells. The human melanoma cell line UACC-62 is included as a reference. Data are represented as means±s.e.m. of two experiments in triplicate. (**d**) Micrographs showing morphological changes driven by shCPEB4 in primary fibroblasts, melanocytes and UACC-62 melanoma cells. Scale bars, 20 μm (unless otherwise indicated). ***P<0.001, *P<0.05. NS, non-significant.

The results above suggested that in contrast to the non-cell autonomous roles of CPEB4 in pancreatic cancer (related to remodelling of the stroma)^35^, this protein could exert a more pressing role in cell proliferation. Therefore, the depletion of CPEB4 was assessed in melanoma cells cultured as monolayers in the absence of other cell types (Fig. 2c). In all cases tested the two CPEB4 shRNAs promoted an acute abrogation of cell proliferation (Fig. 2c), with characteristic features of premature senescence, as determined by staining for β-galactosidase activity at acidic pH (Fig. 2d,e). These effects were in contrast to the minor impact of CPEB4 downregulation in the pancreatic cancer RWP1 (Fig. 3a,b). Similarly, melanoma cells were significantly more dependent on CPEB4 than HeLa, U251 and 639 V (Fig. 3a,b), selected as examples of cell lines from cervical carcinoma, glioblastoma and epithelial sites (bladder cancer), where CPEB4 roles have been best described^4^,^17^,^35^.

We then tested whether the strict dependency of melanomas on CPEB4 was due to the fact that these cells do not express other CPEBs that could act in a compensatory manner. Mining solid tumours in the CCLE (*N*=853; Supplementary Fig. 2a–c), and validating mRNA levels experimentally by qRT-PCR (*N*=20; Supplementary Fig. 3a–c), we found however *CPEB1* and *CPEB2* to be well expressed in melanomas, with levels even higher than for the majority of solid tumours analysed. Levels of *CPEB3* were rather similar in all cell lines studied (Supplementary Figs 2c and 3c; see legends for *P* values of pairwise comparisons to melanoma). Next, we interrogated whether the depletion of CPEB4 in melanoma cells could be counteracted by a compensatory upregulation of other CPEBs. However, qRT-PCR analyses failed to identify changes in the basal levels of CPEBs that would be significantly different upon depletion of CPEB4 in cell lines from melanoma or other tumour types (Supplementary Fig. 3d–g). Altogether, these results point out to distinct roles of CPEB4 in melanoma that (i) cannot be compensated for by other CPEBs and (ii) reflect intrinsic features of this disease that extend beyond the basal levels of CPEBs across tumour types.

### Normal melanocytes reveal lineage-dependent roles of CPEB4

Given the higher sensitivity of melanoma cells to CPEB4 depletion, we decided to address the possibility of lineage-specificity in the requirement of this protein. To this end, CPEB4 was depleted in normal melanocytes, the melanoma precursors. Primary melanocytes were then freshly isolated from biopsies of normal skin and transduced with control and CPEB4 shRNAs. Fibroblasts were also isolated from the same donors for a comparative analysis of genetically matched normal cells of an unrelated lineage. Growth curves were performed with respect to UACC-62 as a prototype of aggressive melanoma cells. Importantly, CPEB4 downregulation (Fig. 3c, upper panels) had rather negligible effects on fibroblasts whereas it reduced the proliferation of melanocytes (Fig. 3c). As the case of melanomas, melanocytes also expressed *CPEB1, CPEB2* and *CPEB3*, but these proteins appear to be unable to compensate for *CPEB4* depletion (Supplementary Fig. 3h). Still, melanocytes maintained their characteristic phenotype without exhibiting the cytoplasmic and nuclear-associated morphological features found in melanoma cells (Fig. 3d).

Overall, these results suggest that melanoma cells may hijack roles of CPEB4 in cell proliferation already present in normal melanocytes. However, melanoma cells appear to acquire additional dependencies on CPEB4 during tumour progression.

### Roles of CPEB4 in melanoma not shared by other cell types

**Figure 4:**
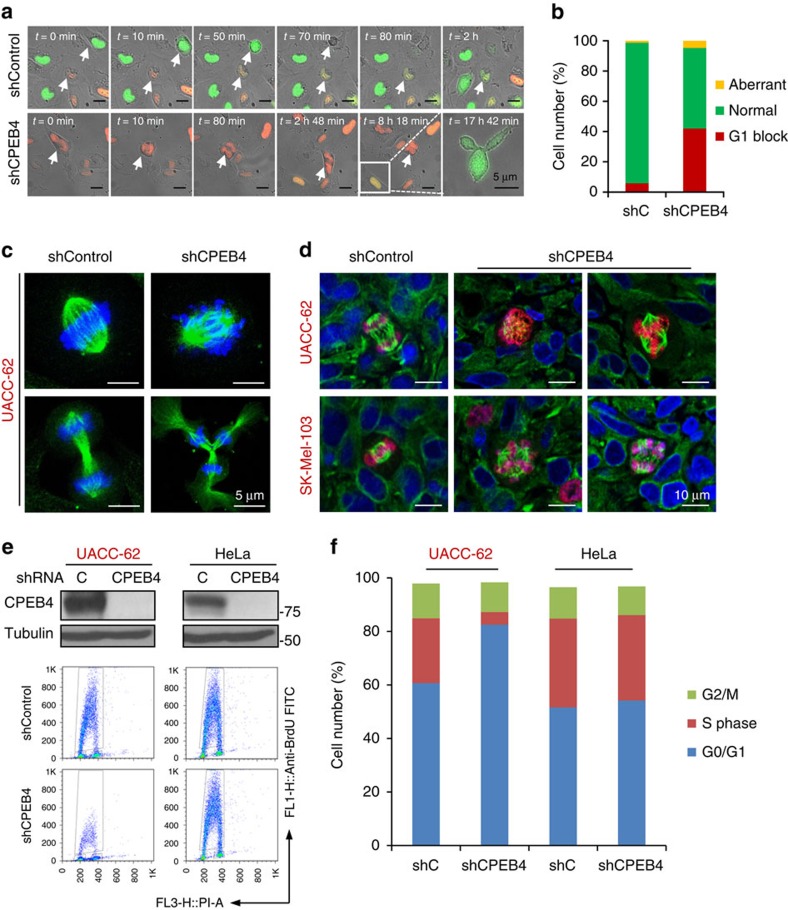
Cell cycle defects in CPEB4-depleted melanoma. (**a**) Time-lapse analysis of aberrant mitosis in cell line SK-Mel-103 labelled with RFP-Cdt1 and GFP-Geminin fusion proteins (FUCCI system)^41^ and transduced with control or CPEB4 shRNAs (see Supplementary Movies 1 and 2 for real time imaging of this process in shC- and shCPEB4-transduced cells, respectively). (**b**) Quantification of cells undergoing normal or aberrant mitosis or blocked in G1 from experiments shown in **a**. (**c**) Confocal imaging of mitotic alterations of cultured UACC-62 melanoma cells expressing control or CPEB4 shRNA. DNA and spindles were visualized by DAPI (blue) and α-Tubulin (green) immunofluorescence, respectively. (**d**) Aberrant mitosis detected by confocal immunomicroscopy in xenografts generated with UACC-62 or SK-Mel-103 cells expressing control or CPEB4 shRNAs. Proliferating cells were identified by phospho-Histone 3 (red immunofluorescence). DAPI (blue) and α-Tubulin (green) were used to label DNA and spindles, respectively. (**e**) Cell cycle profiles of UACC-62 and HeLa cells infected with lentiviruses coding for control or CPEB4 shRNA (see depletion in the immunoblots of the upper panels). Shown are flow cytometry plots of the indicated cells processed to visualize BrdU incorporation. (**f**) Distribution of the indicated cell populations at the different phases of the cell cycle. The percentages of cells at the G0/G1, S or G2/M phases were determined by BrdU and PI staining.

Main known roles of CPEB4 are related to the control of the second meiotic division in oocytes^40^, as well as the exit from mitosis in HeLa^17^ and HEK-293 cells^16^. Therefore, the FUCCI system^41^ was exploited for real-time imaging of cell cycle progression in living melanoma cells expressing or made deficient for CPEB4. This strategy is based on fluorescence-dependent visualization of cells in the G1 phase by means of a RFP-Cdt1 fusion protein (red emission), and cells in S/G2/M by GFP-Geminin (green emission). Real-time videomicroscopy revealed aberrant mitosis visualized by chromosome misalignment in metaphase and aberrant cytokinesis in telophase, after sustained depletion of CPEB4 in melanoma cells (see Fig. 4a–c, as well as Supplementary Movies 1 and 2 for comparative analyses of control- or CPEB4-shRNA cells, respectively). *In vivo*, defects in centrosome number, spindle disorganization, improper alignment of chromosomes and aberrant segregation were also evident by histological evaluation of CPEB4-depleted mouse xenografts (see Fig. 4d for examples of paraffin-embedded lesions stained with α-Tubulin and phosphorylated histone H3, as readouts for spindle formation and the detection of proliferative cells). Single-cell quantifications in cultured cells and subcutaneous melanoma lesions in mice demonstrated that although consistent, mitotic defects in CPEB4-downregulated melanoma cells were infrequent, with <10% increase over basal levels detected in the shControl (shC) cells (Fig. 4b). These results therefore suggested roles of CPEB4 acting at an earlier stage (that is, before mitosis) in the cell division cycle. To evaluate this possibility, cell cycle progression was analysed in greater detail by flow cytometry in melanoma versus non-melanoma lines (UACC-62 and HeLa) transduced with control or CPEB4 shRNAs. As shown in Fig. 4e, BrdU incorporation revealed a distinct decrease in S-phase population of CPEB4-depleted melanoma cells (see quantification in Fig. 4f). Analysis of cell cycle entry after thymidine block confirmed a marked inability to progress through G1-S in melanoma cells, with virtually no changes in the cell cycle profile of the HeLa counterparts (Supplementary Fig. 4). Together, these results demonstrate a new role of CPEB4 in G1/S transition in melanoma superseding a secondary function in mitotic control.

### RIP-Seq identifies new melanoma-enriched CPEB4 targets

**Figure 5:**
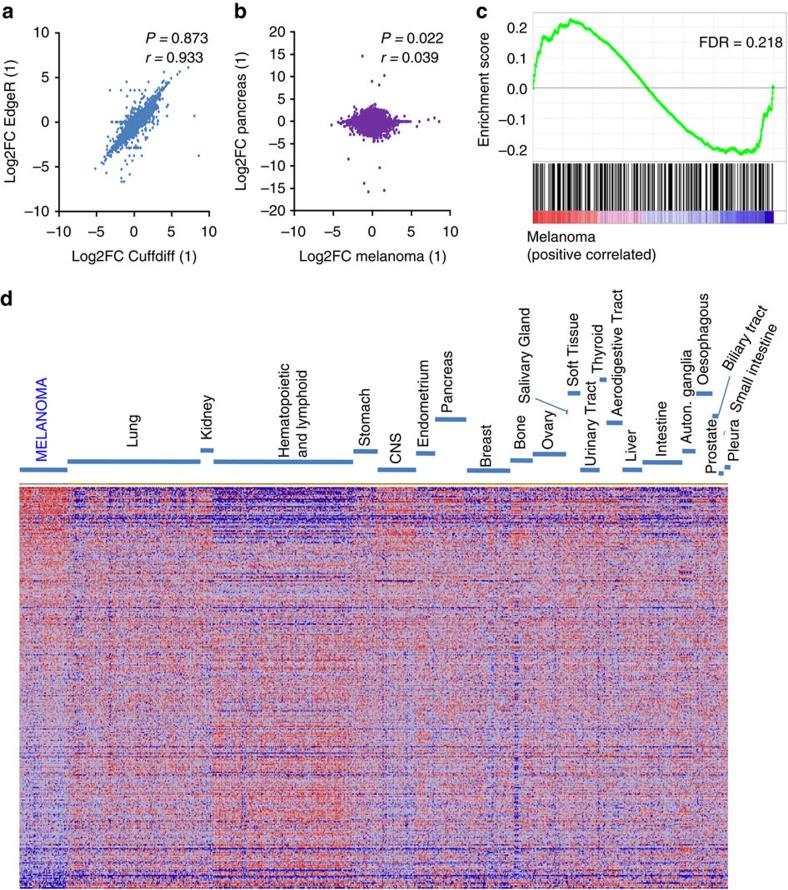
RIP-Seq (RNA immunoprecipitation-sequencing) for the identification of CPEB4 targets in melanoma. (**a**) Correlated results of Cuffdiff or EdgeR-based analysis of CPEB4 RIP-seq analyses in SK-Mel-103, using shCPEB4-derivatives as a reference. This comparison was performed in two independent replicates. Graph depicts differential expression changes (Log2 fold change, Log2FC) obtained with each method for replicate (1). Replicate (2) is shown in Supplementary Fig. 5c. (**b**) Differential expression of CPEB4-bound mRNAs in SK-Mel-103 versus the RWP1 pancreatic cancer cell line. RIP-seq data from RWP1 was obtained from ref. 35 and analysed as for melanoma cells by Cuffdiff. Two replicates were processed for each cell line. Data in this panel correspond to Replicate (1) of melanoma and pancreatic cancers. Other replicates are depicted in Supplementary Fig. 5d. (**c**) Relative expression of CPEB4-bound mRNAs identified by RIP-seq in SK-Mel-103 cells and mined by GSEA across the CCLE data set. Graph represents the enrichment score in melanoma versus other tumours. Positive correlated genes in melanoma and negatively correlated in other tumours are highlighted in red and blue, respectively. (**d**) Heatmap from the GSEA analysis shown in **c**, represented for each of the indicated tumour cell types. Note the distinct clustering in melanoma. Pearson coefficient (P), Spearman rank correlation coefficient (r) and FDR values are indicated in the corresponding panels.

Next, RNA immunoprecipitation followed by RNA sequencing (RIP-seq) was performed for an unbiased identification of CPEB4-bound transcripts that are involved in the modulation of mitosis and the lineage-specific control of G1/S transition. In this context, we were interested in genes or pathways that may act beyond the control of pigmentation (that is, to identify true novel melanoma-associated traits). The melanoma SK-Mel-103 was selected for an initial screen, as a well-known example of MITF-negative amelanotic melanoma cells^31^. RNA collected from control or shCPEB4-transduced cells (two independent replicates each) was subjected to crosslinking and immunoprecipitation followed by elimination of ribosomal RNA (total reads are summarized in Supplementary Fig. 5a). Reads were aligned to the human genome (Ref Seq GRCh37/hg19) with TopHat-2.0.4 (ref. 42). For each read, we considered the best hit or allowed 20 multi-hits, obtaining an 80–85% overlap with the two approaches (Supplementary Fig. 5b). We thus proceeded with the best hit for subsequent analyses of differential expression. This was performed using Cufflinks or EdgeR, which rendered similar results as indicated by the high correlation found by Pearson and Spearman rank tests (Fig. 5a; see also Supplementary Fig. 5c). Filtering for significance (adjusted *P* value<0.05), this approach rendered 331 CPEB4-bound transcripts in melanoma cells (that is, downregulated in the CPEB4 shRNA counterparts; see additional detail in Supplementary Data 1). Similar analyses were performed for CPEB4 RIP-Seq in the pancreatic RWP1 cell line (which had also been performed in duplicates)^35^. Intriguingly, the overlap between RIP-Seq data of both systems was strikingly low (see Fig. 5b for one of the replicates of SK-Mel-103 and of RWP1, and Supplementary Fig. 5d for the rest of the comparisons). Thus, while known targets of CPEB4 such as the metallothionein proteins *MT2A* and *MT1E* were found in both cell types, 93% of the CPEB4-bound transcripts in melanoma (that is, 312/331) had not been reported in RWP1 (Supplementary Fig. 5e). Indeed, Gene Set Enrichment Analyses (GSEA) performed in the CCLE, indicated the CPEB4-bound targets in melanoma showed a distinct expression in this tumour type (see enrichment scores in Fig. 5c; the corresponding heatmaps in Fig. 5d; false discovery rate (FDR)=0.22). Therefore, these results support the hypothesis that CPEB4 has functions restricted to melanoma resulting (at least in part) from the regulation of a set of genes particularly enriched in this tumour type.

**Figure 6:**
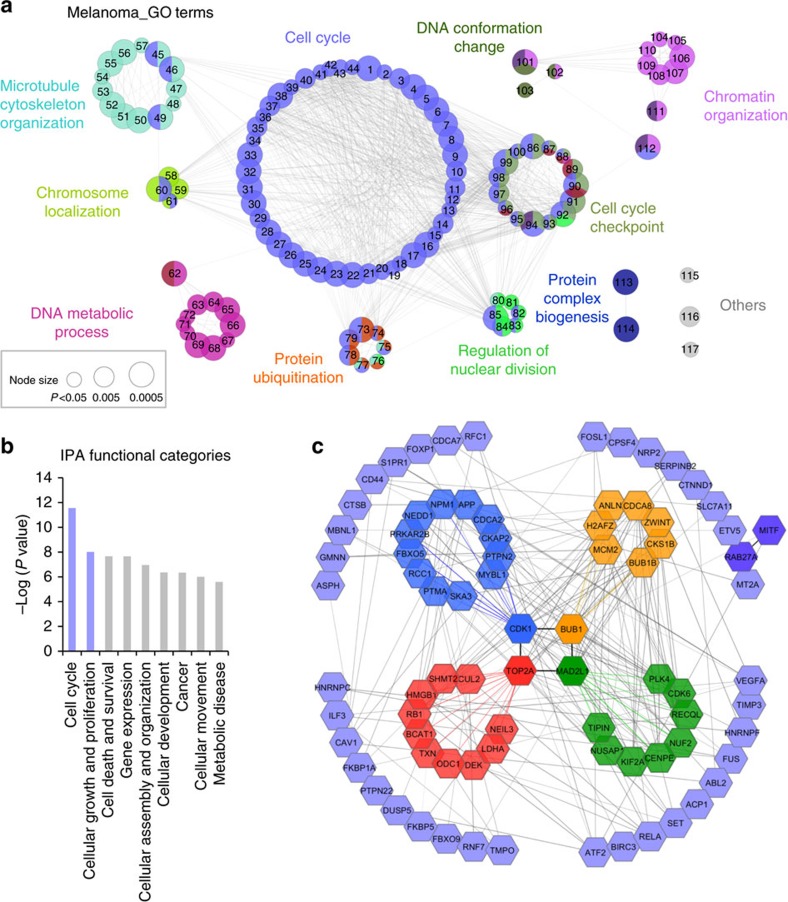
RIP-Seq identifies cell cycle regulators and lineage-specific oncogenes as novel CPEB4 targets. (**a**) Interaction networks of the GO-terms (database 02.10.2015) enriched in the CPEB4-bound transcripts identified by RIP-seq in SK-Mel-103. Data were plotted using Cytoscape v3.2.1 and the ClueGO plug-in v2.1.7 (see GO-terms networks of RWP1 pancreatic cell line in Supplementary Fig. 6a). Numbers correspond to GO-gene sets further described in Supplementary Data 2. Node sizes represent the statistical significance of the terms (***P<0.0005; **P<0.005; *P<0.05). Supplementary Data 2 contains detailed information on the CPEB4-bound transcripts and the specific genes in the 117 identified clusters. Supplementary Data 3 lists GO-enriched categories for CPEB4-bound targets in RWP1 pancreatic cancer cells to demonstrate the minimum overlap with the melanoma SK-Mel-103. (**b**) IPA of functional categories enriched in CPE-containing transcripts identified by CPEB4 RIP-Seq in SK-Mel-103. (**c**) Protein–protein interaction network of the genes included in the IPA ‘cell cycle' and ‘cellular growth and proliferation' clusters of **b** analysed by STRING and Cytoscape for the visualization of signalling hubs recognized by CPEB4 in melanoma cells (see Supplementary Fig. 7 for validation of selected CPEB4 direct targets).

Data from RIP-Seq generated in SK-Mel-103 and RWP1 were further analysed to better identify biological processes that may ultimately account for the strict requirement of CPEB4 in melanocytic cells. Using Cytoscape and the ClueGO plug-in for network visualization, 117 gene ontology (GO) clusters were identified in SK-Mel-103 (*P-*values corrected for multiple testing <0.05; see Supplementary Data 2 for detail). The most represented GO term was ‘Cell Cycle', with 44 highly interrelated GO-associated sub-clusters (see Fig. 6a). Related interconnected networks were (i) ‘Cell Cycle Checkpoints', (ii) ‘Chromatin Organization and DNA Conformation Change', (iii) ‘DNA Metabolism' and (iv) ‘Microtubule Cytoskeleton Organization', among others. Intriguingly, these gene clusters showed a minimum overlap with the GO-enriched terms in pancreatic cancer cells (Supplementary Fig. 6a). Specifically, CPEB4 was found to bind transcripts of some mitotic genes (such as *BIRC5* or *CENPU*) in pancreatic cells, but the majority of the enriched functions were related to metabolism, ribosome biogenesis, macromolecule localization and organization, intracellular transport and various catabolic processes (Supplementary Fig. 6b; Supplementary Data 3), consistent with previous roles of CPEB4 in the secretion of pro-metastatic factors^35^. Therefore, these results significantly expand targets and functions of CPEB4. Moreover, the lack of concordance between melanoma and pancreatic cancer cells may account for the differential dependency of these cells on CPEB4 described above (Figs 3 and 4e,f).

### PAT-assays validate CPEB4 targets enriched in melanoma cells

**Figure 7:**
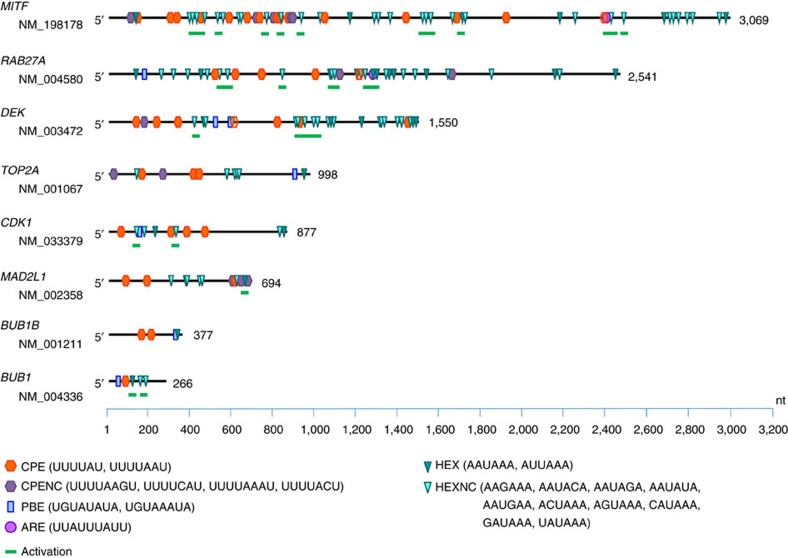
3′-UTR map of CPEB4 targets. Schematic representation of binding sites for cytoplasmic polyadenylation-associated factors located at the 3′-UTR of selected CPEB4 targets identified by RIP-seq in melanoma cells. The content and combination of these binding sites were identified and analysed by the customized algorithm described in ref. 43 for the prediction of their potential regulation by cytoplasmic polyadenylation. ARE, AU-rich element; CPE, cytoplasmic polyadenylation element; CPENC, non-consensus CPE; HEX, polyadenylation hexanucleotide; HEXNC, non-consensus hexanucleotide; PBS, Pumilio-binding site.

The 331 CPEB4-bound transcripts identified by RIP-seq in melanoma were subsequently filtered by customized prediction algorithms for the identification of transcripts with optimally positioned CPEs in the 3′-untranslated region (UTR)^43^. Specifically, these computational methods take into account the number and localization of CPEs (5′-UUUUA_1-2_U) with respect to polyadenylation hexanucleotides (HEX), AU-rich elements (ARE) and binding sites for the Pumilio RNA-binding protein (PBS), all influencing competency for cytoplasmic polyadenylation^43^ (see Supplementary Methods and Fig. 7 below for additional information on the specific sequences analysed, which include also non canonical variants of CPE and HEX). This computational analysis resulted in 226 CPEB4-bound mRNAs with *bona fide* CPEs (Supplementary Data 4a). These CPE-containing mRNAs were further analysed using Ingenuity Pathway Analysis (IPA) (Fig. 6b). This revealed a narrower set of genes in ‘Cell Cycle' and ‘Cell Growth and Proliferation' categories (corrected *P* value=2.75 × 10^−12^ and *P*=9.75 × 10^−9^, respectively, Fig. 6b; see complete gene lists in Supplementary Data 4b–d). Interestingly, although the number of genes in these categories was large (*N*=106), they were cohesively interconnected around four nodes, which were found to centre on BUB1, CDK1, TOP2A or MAD2L1, classical mitosis checkpoint controllers (Fig. 6c). Linked to these factors were also multiple key cell cycle regulators (for example, BUB1B), and oncogenes such as the chromatin remodeler DEK, which we had previously reported with key roles in the maintenance of the proliferative capacity of melanoma cells^44^,^45^. All these genes contained multiple CPEs, HEX and PBS in their 3′-UTR (Fig. 7), as expected for transcripts regulated by cytosolic polyadenylation^43^.

It would be informative, albeit not practicable, to validate each of the 106 transcripts mentioned above as direct CPEB4 targets. Therefore, to gauge the strength of this gene list, we selected BUB1B, CDK1 and DEK for validation of mitosis CPEB4-related oncogenic hubs. The requirement of CPEB4 to maintain the expression of BUB1B, CDK1 and DEK was confirmed by protein immunoblotting after shRNA depletion in three cell lines from melanoma (SK-Mel-28, SK-Mel-103 and UACC-62), using other three non-melanoma lines (RWP1, HeLa and U251) as a reference (Supplementary Fig. 7a; note the modest or inconsistent downregulation of the analysed genes by CPEB4 depletion in the non-melanoma lines with respect to the downregulation in the melanoma cells). Furthermore, additional RNA-immunoprecipitations demonstrated binding of CPEB4 to the 3′-UTR of *BUB1B*, *CDK1* and *DEK* mRNAs in melanoma cells (Supplementary Fig. 7b–d). In addition, poly(A) tail tests (RNA-ligation-coupled RT-PCR) confirmed the shortening of the poly(A) tail and the destabilization of these three mRNAs upon CPEB4 depletion in melanoma, but not in other cell types (Supplementary Fig. 7e). Together, these results emphasize a distinct impact of CPEB4 in the regulation of key mitosis-associated oncogenic networks in melanoma.

### CPEB4 controls lineage-specific melanoma drivers

**Figure 8:**
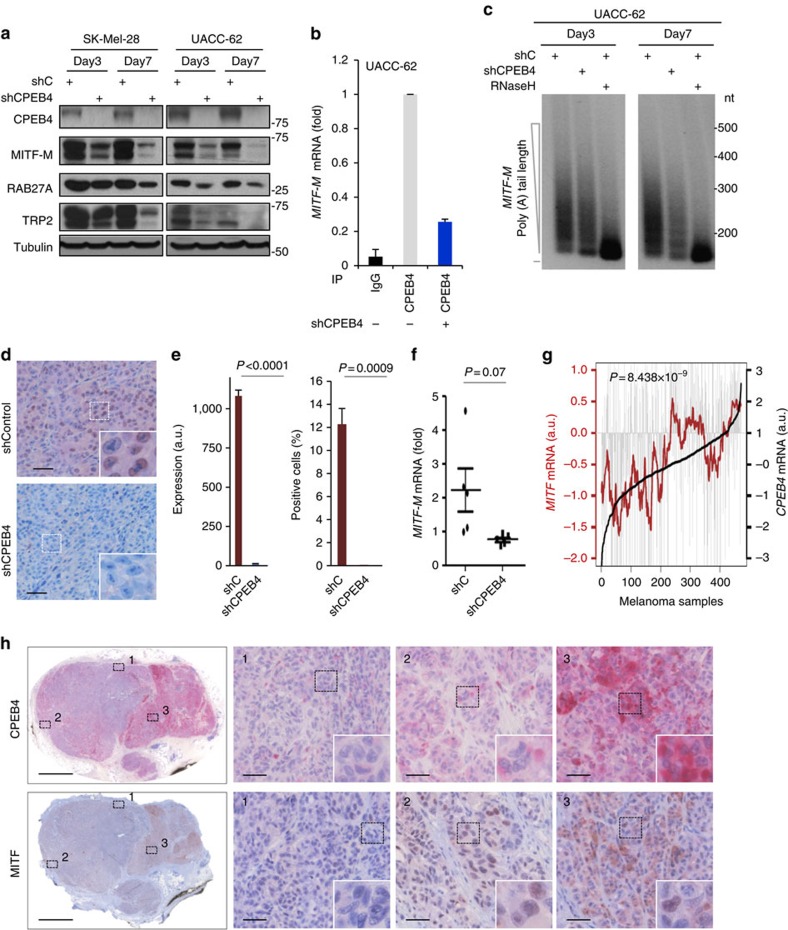
CPEB4-driven control of the lineage-specific transcription factor MITF. (**a**) Impact of CPEB4 depletion (by shRNA) on the protein levels of MITF and the indicated targets shown by immunoblotting in two independent melanoma cell lines. (**b**) Relative levels of *MITF* mRNA immunoprecipitated with antibodies for CPEB4 or rabbit IgG in UACC-62. Cells transduced with shCPEB4 were set as a reference for specificity. mRNA levels were normalized against expression in the inputs (that is, parental or shCPEB4-expressing cells) and are represented as means±s.e.m. from triplicates. (**c**) PAT (polyadenylation length test) of *MITF 3*′-U*TR* in shC or shCPEB4-transduced melanoma cells. RNase H was used for poly(A) tail removal to define the specificity of the amplification procedure as previously described^35^. (**d**) Paraffin embedded sections of mouse xenografts generated with UACC-62 expressing shC or shCPEB4, and processed for immunohistochemical detection of MITF protein (brown signal). Nuclei were co-stained by hematoxylin. Scale bars, 50 μm. (**e**) Bar graphs depicting the staining intensity and fraction of MITF-positive cells per section in xenografts generated as in **d**. Data are represented as means±s.e.m. from *N*=5 tumours per group. (**f**) Relative expression of *MITF* mRNA in xenografts generated as in **d** determined by quantitative qRT-PCR. (**g**) *MITF* and *CPEB4* mRNA expression in 471 melanoma specimens from the TCGA database. Melanomas are ranked by expression of *CPEB4* (from left to right), which is represented by the black line. Grey lines indicate *MITF* expression in each sample and the moving average is represented by the red line. *P* value of Spearman correlation analyses is indicated. (**h**) Immunohistochemical detection of CPEB4 (pink) and MITF (brown) in consecutive sections of a representative metastatic melanoma specimen. Shown are three areas with low (1), intermediate (2) and high (3) CPEB4 staining. Note the parallel expression of both proteins. Stainings were repeated in five independent specimens obtaining similar results. Scale bars, 200 μm (left images); and 50 μm (right images). Student's *t*-test *P* values (*P*) are indicated in the corresponding panels.

**Figure 9:**
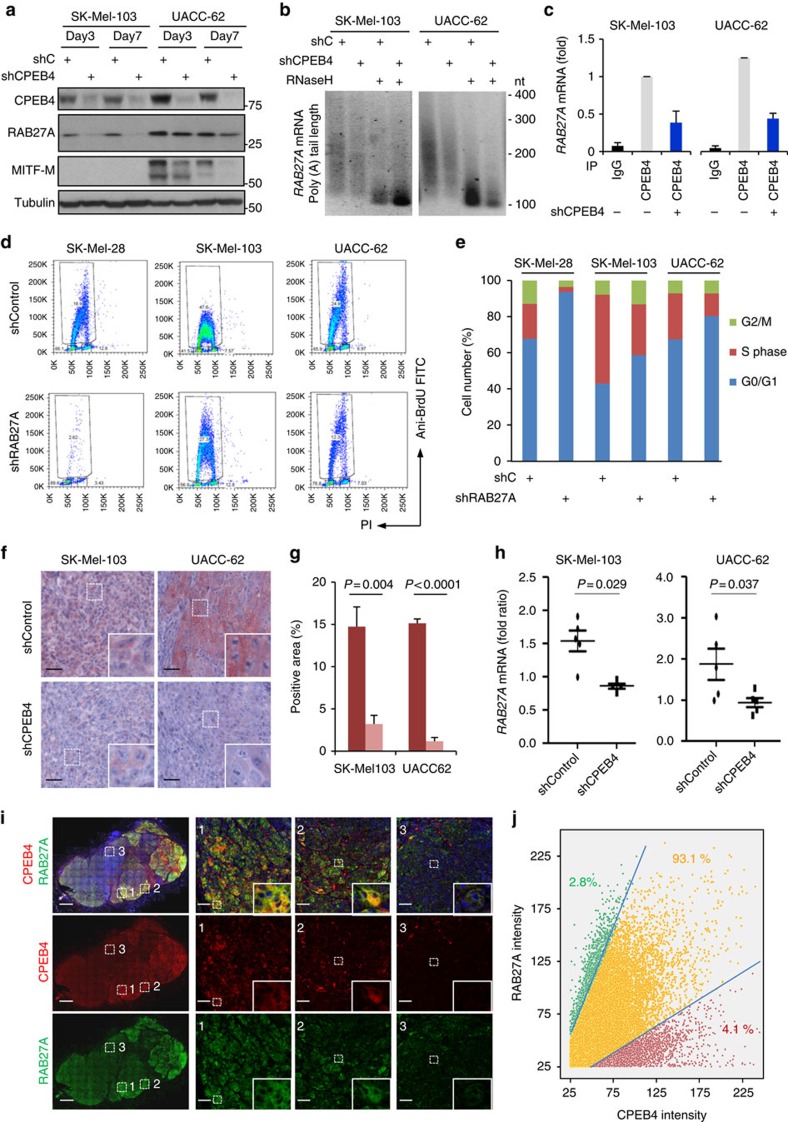
RAB27A as a novel CPEB4-controlled melanoma driver. (**a**) Immunoblots showing the downregulation of RAB27A protein expression in MITF-negative (SK-Mel-103) and MITF-positive (UACC-62) cell lines at the indicated times upon lentiviral-driven expression of control or CPEB4 shRNA. (**b**) *RAB27A* mRNA Poly(A) tail shortening visualized by PAT assays in CPEB4-depleted melanoma cells. RNase H was used as a reference control for poly(A) removal. nt, nucleotides. (**c**) *RAB27A* mRNA levels from RIP experiments performed with CPEB4 antibody or IgG control antibody in the indicated melanoma cells. Inputs were used to normalize mRNA expression in the immunoprecipitated fraction and data are presented as means±s.e.m. from triplicates. (**d**) BrdU incorporation in the indicated melanoma cell lines visualized by flow cytometry 4 days after lentiviral-driven expression of control or RAB27A shRNA. The corresponding cell cycle distribution is shown in **e**. (**f**) Micrographs of paraffin-embedded sections of xenografts generated with the indicated cell lines expressing shC or shRNA against CPEB4, and processed for the visualization of RAB27A (pink staining). Nuclei are counterstained with hematoxylin. Scale bars, 50 μm. (**g**) Quantification of RAB27A expression represented as a function of positive cells. (**h**) *RAB27A* mRNA downregulation determined by quantitative qRT-PCR in xenografts generated as in **f**. (**i**) Mosaic image corresponding to dual immunohistochemistry performed on human melanoma tumours (whole-lesion analysis) and visualized by confocal microscopy for single-cell quantification of CPEB4 (red) and RAB27A (green). Scale bars, 1,000 μm. Images in the right correspond to higher magnification of three selected areas of the lesion (labelled as 1, 2 and 3; scale bars, 100 μm) demonstrating the correlation between these two proteins. (**j**) Relative expression of CPEB4 and RAB27A quantified at a single-cell level by an intelligent matrix screening remote control tool (iMSRC) from images processed by the Definiens XD software. Data points were pseudo-coloured to separate cells with dual expression of CPEB4 and RAB27A (yellow) from those with dominance of one of the two proteins (green for RAB27A and red for CPEB4). Student's *t*-test *P* values (*P*) are indicated in the corresponding panels. iMSRC, intelligent matrix screening remote control.

Downregulation of BUB1B, CDK1 and DEK provides a mechanistic explanation for the aberrant spindles and defects in cytokinesis observed after sustained depletion of CPEB4 in melanoma cells (Fig. 4a–d). However, these genes cannot account for the acute and melanoma-enriched G1 arrest after CPEB4 downregulation, and could not explain why melanomas would be more dependent on this polyadenylation factor than other tumour types. The CPEB4 RIP-seq networks mentioned above in SK-Mel-103 (Fig. 6b,c) were then further screened for genes with unique functions in melanoma. This identified RAB27A (Fig. 6c), a vesicle trafficking modulator known for its essential roles in melanosome maturation (a defining trait of this cell lineage)^46^. Moreover, RAB27A is upregulated during melanoma progression and it is required for cell division, although by still incompletely defined mechanisms^22^. Although not expressed in SK-Mel-103, we questioned whether CPEB4 could also control an upstream modulator of *RAB27A*, the transcription factor MITF. MITF was relevant as the prototypical lineage-specific oncogene in melanocytic tumours^47^. Interestingly, we found the *MITF* 3′-UTR to contain the largest amount of CPEs, HEX and PBS of all the genes analysed, which as the case for *RAB27A* were predicted by our computational models to be optimally organized for the control by cytoplasmic polyadenylation (Fig. 7). Therefore, we set to test the functional impact of CPEB4 in the levels and polyadenylation status of both, *MITF* and *RAB72A* in additional melanoma cell lines and in tissue specimens (Figs 8 and 9, respectively).

To demonstrate the impact of cytoplasmic polyadenylation on MITF, CPEB4 was downregulated in SK-Mel-28 and UACC-62 (two representative pigmented melanoma cell lines) using validated CPEB4 shRNAs (Fig. 8a). In both cases, the absence of CPEB4 resulted in a marked inhibition of MITF-M, the most abundant MITF isoform in melanoma cells^48^, as shown in Fig. 8a.

Next, RIP-qPCR and PAT-assays were performed to confirm direct binding of CPEB4 to the *MITF* 3′-UTR and the subsequent poly(A) shortening CPEB4 depletion (see Fig. 8b,c). MITF protein and mRNA were then assessed in mouse xenografts generated with control or CPEB4-transduced shRNAs. As shown in Fig. 8d,e, downregulation of CPEB4 resulted in a marked reduction of MITF protein. Further supporting shortened poly(A) tail affecting transcript stability (Fig. 8c), *MITF* mRNA levels were relatively lower in CPEB4 depleted versus parental mouse xenografts (Fig. 8f). To independently validate MITF as *bona fide* target of CPEB4, two short guide RNAs were generated for CRISPR/Cas9-mediated gene depletion. As shown in Supplementary Fig. 8a,b, CPEB4 sgRNAs promoted a marked inhibition of MITF in pigmented melanoma cells with the consequent blockade of cell proliferation.

To further address the physiological impact of these results, the Cancer Genome Atlas (TCGA) was mined to assess the relative expression of *CPEB4* and *MITF* in human melanoma specimens (*N*=441). As shown in Fig. 8g, a positive correlation was indeed found in mRNA levels of both genes (*P*=8.438 × 10^−9^). In parallel, immunohistochemical analyses in representative examples of human melanoma lesions showed that although MITF protein expression was highly variable as expected^25^,^49^, it was also positively correlated with CPEB4 levels (see areas with low, intermediate and high levels of both CPEB4 and MITF in Fig. 8h). Interestingly, depletion assays demonstrated that the requirement of CPEB4 to maintain MITF levels was also found to extend to normal melanocytes, the cells or origin of melanomas (Supplementary Fig. 8c). Together, these results link for the first time CPEB4 to the regulation of inherent features of melanocytic lineage.

### RAB27 as a fail-safe control of melanoma cell proliferation

As mentioned above, *RAB27A* is a well-known transcriptional target of MITF. However, additional mechanisms of regulation of RAB27A may exist as this gene can maintain its expression despite multiple post-translational events that can dampen MITF activity^26^,^27^,^28^,^29^. Therefore, since we had identified *RAB27A* as a CPEB4-binding transcript (Fig. 6c), we questioned whether cytoplasmic polyadenylation could sustain the expression of RAB27A (and thus, its functions in cell proliferation) even in the absence of MITF. To this end, CPEB4 was downregulated by lentiviral-driven transfer of shRNA, or by two guide RNAs for CRISPR/Cas9 gene depletion (Fig. 9a and Supplementary Fig. 8a,b) in MITF-negative and -positive cell lines (SK-Mel-103 and UACC-62, respectively). In both cases (particularly for MITF-deficient melanoma cells) RAB27A levels were reduced in the absence of CPEB4 (Fig. 9a and Supplementary Fig. 8a,b). Of note, normal melanocytes were also found dependent on CPEB4 to sustain RAB27A expression (see Supplementary Fig. 8c for inhibitory effects of CPEB4 shRNA on these cells determined by immunoblotting).

Next, PAT-assays were used to demonstrate that CPEB4 indeed modulated *RAB27A* mRNA polyadenlyation. As shown in Fig. 9b, *RAB27A* poly(A) tail was shortened after CPEB4 depletion, downstream of binding to the 3′-UTR of *RAB27A*, as supported by amplification of this region upon RNA immunoprecipitation (Fig. 9c). Functionally, as the case for CPEB4, the reduction of RAB27A expression (mimicked with a validated shRNA) also promoted G1 arrest (see flow cytometry analyses in Fig. 9d,e). Therefore, these results identify polyadenylation as a new mechanism of regulation of RAB27A, distinct from the classical transcriptional control by MITF^50^, and the GDP-GTP exchange characteristic of small GTPases^51^.

A corollary of the data above is that RAB27A and CPEB4 expression should be tightly correlated *in vivo*. Histopathological examination of mouse subcutaneous xenografts generated with control or CPEB4-shRNA transduced melanoma cell lines showed a marked reduction of RAB27A protein (see Fig. 9f,g, for matched pairs of CPEB4 positive and negative lesions generated with two independent cell lines and the corresponding quantifications). RNA extraction from these CPEB4-deficient murine lesions confirmed the expected reduction in *RAB27A* mRNA (Fig. 9h). These results were further confirmed in human metastatic melanomas. Immunofluorescence imaging by confocal microscopy of human melanomas (Fig. 9i), followed by single-cell quantification analyses in whole-tissue sections (Fig. 9j) revealed that over 90% of cells co-express both proteins (Pearson correlation *P*=0.69). Therefore, the histological and functional results of this study bring physiological relevance to our initial computational data which had revealed a particular enrichment of *CPEB4* in melanoma (Fig. 1a), also characteristic for *RAB27A* (Supplementary Fig. 8d). Specifically, here we show that by overexpressing CPEB4, melanoma cells can ensure an efficient progression through the cell cycle (through newly identified gene networks that act at G1/S and G2/M checkpoints), while maintaining lineage-specific identity via an independent control of MITF and RAB27A.

## Discussion

**Figure 10:**
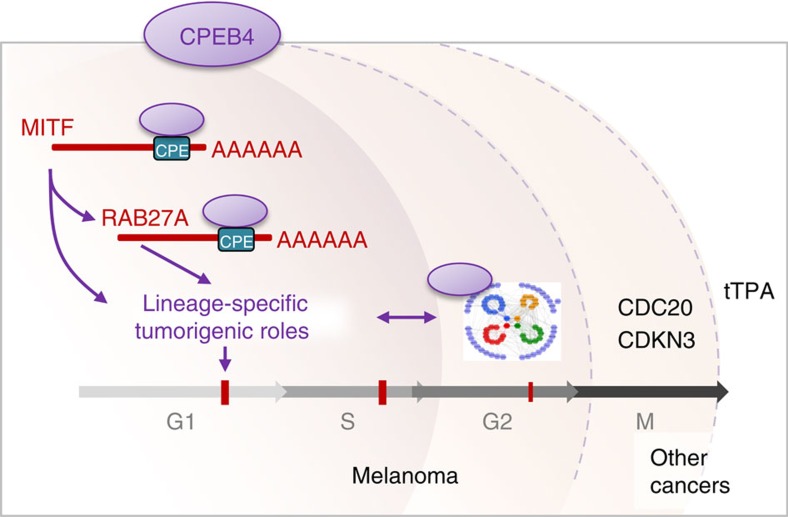
Proposed mode of action of CPEB4 in melanoma. Summary of newly identified roles of CPEB4 on lineage-specific melanoma drivers (MITF and RAB27), superseding a cohesive network of G2/M cell cycle modulators, both distinct from targets described in pancreatic cancer (the only other tumour type where CPEB4 function has been analysed as here, in a genome-wide manner).

Lineage-specificity in cancer can be achieved by genes that are either uniquely expressed in precursor cells (usually as part of signalling cascades that define cell identity), or by factors that being ‘common' to other malignancies, are distinctively regulated and required for a selected tumour type^52^,^53^. Here we demonstrate that in melanoma cells, these two mechanisms can be coordinately controlled by CPEB4: (i) by modulating the expression of MITF and RAB27A, essential drivers of intrinsic melanocytic-associated functions; and (ii) by enriching the expression of key oncogenic signalling hubs (see model in Fig. 10). These data therefore provide new insight on mechanisms underlying melanoma progression and uncover unexpected tumour-specific actions of cytoplasmic polyadenylation in this disease.

Melanomas deregulate a broad spectrum of (epi)genetic alterations which ultimately impinge at multiple levels on cell cycle checkpoint controls^36^,^54^,^55^,^56^,^57^. Consequently, it could perhaps have been anticipated that melanomas behaved in a similar manner as aggressive pancreatic cancers, namely, being able to sustain *CPEB4* depletion without affecting cell cycle progression^35^. Indeed, our data revealed that melanomas -as other tumour types- express other CPEB family members (particularly *CPEB1* and *CPEB2*), which could have acted in a compensatory manner in the absence of CPEB4. Yet, one of the most unexpected findings of this work was the acute abrogation of cell proliferation in CPEB4-downregulated melanoma cells (a feature we could trace back to normal melanocytes). The minimal overlap in the CPEB4-bound transcripts in melanoma and pancreatic cancers is striking, considering that up to 20% of the transcriptome has been proposed to be controlled by cytoplasmic polyadenylation^17^. Therefore, the extent to which CPEBs may substitute for or cooperate with CPEB4 in other tumour types deserves further attention. Furthermore, it should be taken into consideration that CPEBs may act as activators or repressors of translation depending on their binding partners and cellular context^9^. For example, an attractive possibility is that the RNA maps controlled by CPEB4 are wired in a differential manner depending on whether this protein is overexpressed or downregulated. These studies may be further complemented with the analysis of 3′-UTRs with non-canonical CPE sites, which may also be regulated by CPEB4 as suggested by our RIP-Seq data.

The G2/M transition modulators identified as CPEB4 targets in melanoma provide a mechanistic explanation for the aberrant mitosis observed *in vitro* and *in vivo* upon depletion of this protein. In this context, transcripts associated with spindle assembly and chromosome segregation found here for CPEB4 in melanoma reflect mitotic roles of CPEB1 first described in *Xenopus* oocytes^58^. These results therefore support an attractive scenario whereby CPEB4 may have acquired pro-mitotic functions of CPEB1. However, our data suggest that in melanoma, this function is superseded by factors that act earlier in the cell cycle (that is, G1/S transition), and have essential roles in this tumour type (see model in Fig. 10). Here we showed that this dependency relies in a cluster of genes selectively enriched in melanoma, a feature we functionally demonstrated for MITF and RAB27A. CPEB4 may therefore endow these melanoma-associated factors for fast tuning, polyadenylating their mRNA in the cytosol and favouring translation without the time and energy needed for new transcription, splicing and exit from the nucleus. This scenario may reflect actions of CPEBs on other cell cycle regulators such as Cyclin B, Cyclin E1 or E2 in oocytes^40^. It would be interesting to determine whether transcriptional roles of MITF could in turn contribute to the upregulation of CPEB4 at early stages of melanoma progression, particularly in the context of classical oncogenic alterations in this disease (for example, BRAF>MEK or NRAS>PI3K-dependent pathways).

The finding that CPEB4 can control RAB27A independently of MITF has further translational implications. RAB27A was initially identified for its role in melanosome biogenesis and transport^50^. However, this protein, as other vesicular trafficking modulators, is raising attention as part of an oncogenic signature we and others have found to be particularly hyperactivated in melanoma^22^,^31^,^32^,^33^. The presence of CPE sites in the *RAB27A* 3′UTR therefore allows the regulation by polyadenylation to secure a ‘melanocytic memory' even in the absence of MITF. Consequently, CPEB4-controlled pathways could represent cell type-selective vulnerabilities that could be exploited therapeutically. Of note, while this study has focused on melanocytic cells, *CPEB4* is expressed (albeit to variable levels) in other cell types. Therefore, it is tempting to speculate that CPEB4 may polyadenylate other RAB family members that modulate protein sorting or secretion for example to modulate neuronal synaptic plasticity or cell polarity, processes reported to be controlled by polyadenylation^4^. Therefore, the RNA immunoprecipitation analyses performed here provide a rich resource on CPEB4 controlled signalling cascades that could be explored as a platform for future comparative analyses across tumour types and cellular lineages.

## Methods

### Cells

The human melanoma cell lines SK-Mel-5, SK-Mel-19, SK-Mel-28, SK-Mel-29, SK-Mel-103, SK-Mel-147, SK-Mel-173, G-361, UACC-62 were obtained from the Memorial Sloan Kettering Cancer Center, NY, USA and WM-164 from Dr Herlyn's laboratory at Wistar Intitute, PE, USA. The non-melanoma human cell lines 639 V (bladder cancer), HeLa (cervical cancer), HCT116 (colorectal cancer), T98G (glioblastoma), U251 (glioma), A549 (non-small cell lung cancer), PC3 (prostate cancer) and 293FT (transformed human embryonic kidney cells) were obtained from ATCC. MiaPaca-2 and RWP1 (pancreatic cancer), as well as HT29 (colorectal cancer) were a gift from Dr F Real's laboratory at CNIO. All these tumour cell lines were cultured in DMEM (Invitrogen) supplemented with 10% fetal bovine serum (Hyclone). Primary human melanocytes and fibroblasts were isolated as described before^59^ from neonatal foreskins. Melanocytes were cultured in Medium 254 supplemented with 1% melanocyte growth factors (HMGS, Cascade Biologics) and 0.2 mM CaCl_2_; fibroblasts were cultured in DMEM containing 10% fetal bovine serum. All primary and tumour cell lines were mycoplasma free as tested by nucleic acid hybridization-based assays.

### Protein immunoblotting

Cells were harvested and total cell lysates were obtained using RIPA buffer supplemented with protease (Roche Diagnostics) and phosphatase inhibitors (Santa Cruz). Protein immunoblots were performed according to standard procedures using Immobilon-P membranes (Millipore). Primary antibodies used were: CPEB4 (ab830009, Abcam; dilution 1:1,000), DEK (610948, BD Transduction Laboratories; dilution 1:1,000), BUB1B (612503, BD Transduction Laboratories; dilution 1:500), CDK1 (sc-54, Santa Cruz Biotechnology; dilution 1:500), RAB27A (HPA001333, Sigma; dilution 1:1,000), MITF (Ab-1, Clone C5, Thermo Scientific; dilution 1:500), TRP2 (sc-54, Santa Cruz Biotechnology; dilution 1:1,000) and α-Tubulin (clone DM1A, Sigma; dilution 1:10,000). HRP-conjugated secondary antibodies (dilution 1:5,000) were either anti-mouse (GE Healthcare), anti-rabbit (GE Healthcare) or anti-goat (Jackson Immunoresearch). When indicated, ImageJ software was used to quantify protein levels. α-Tubulin was used as loading control. Uncropped scans of the most important blots are provided in the Supplementary Information (Supplementary Figs 9–11).

### Gene silencing by lentiviral transduction of shRNAs

Stable depletion of CPEB4 was achieved by lentivirus-driven gene silencing using two previously reported shRNAs^35^ purchased from Sigma: CPEB4 sh1: 5′-GCTGTTGGAAAGACTTGATAA-3′ and CPEB4 sh2: 5′-GCGTTATGTGTTGAACAGTAT-3′. Non-Target shRNA: 5′-CAACAAGATGAAGAGCACCAA-3′ was used as control. Viruses were produced in 293FT cells and infections were performed as previously described^60^. Downregulation efficacy was determined after puromycin selection (1 μg/ml) by protein immunoblotting or RT-qPCR.

### Growth curves and colony formation assays

For growth curves, 1 × 10^3^ tumour or 5 × 10^3^ primary cells were plated in 96-well optical bottom plates at day 6 after lentiviral transduction. At the indicated time intervals, cells were fixed with 4% paraformaldehyde (Electron Microcopy Sciences) and stained with DAPI (Invitrogen). For each time point, total cell number was quantified in triplicates by automated high-throughput confocal detection of DAPI-stained nuclei using the OPERA HCS platform and the Acapella Analysis Software (Perkin Elmer). Colony formation assays were performed on six-well plates, each well seeded with 5 × 10^4^ cells for high density assays or 2–4 × 10^3^ cells for low density assays onto six-well plates. Cells were allowed to grow for 7–14 days and the colonies were then stained with 0.4 g l^−1^ crystal violet (Sigma). Crystal violet intensity was quantified using ImageJ software. Unless otherwise indicated, all proliferation and colony formation assays performed with lentiviral transduced cells were plated at day 6 post transduction. The number of biological and technical replicates for each experiment is indicated in the figure legends.

### Cell cycle progression analyses

For BrdU pulse, exponentially growing UACC-62 and HeLa cells transduced with control- or CPEB4- shRNAs were incubated with 10 μM BrdU (Sigma) for one hour, essentially as described elsewere^61^. S phase (BrdU positive) cells were stained with FITC-conjugated anti-BrdU antibody (BD Pharmigen) and DNA was counterstained with 50 μg ml^−1^ propidium iodide (PI) (Sigma). For cell synchronization at the G1/S phase, UACC-62 and HeLa cells were treated with 2.5 mM thymidine (Sigma) for 19 h, starting one day after transduction with control or CPEB4 shRNAs. Cells were then fixed at 0, 4, 8 and 24 h after release of the thymidine block and DNA counterstained with 50 μg ml^−1^ PI (Sigma). Data were acquired using a FACS Calibur flow cytometer (Becton Dickinson). Cell aggregates were excluded using pulse processing and a minimum of 20,000 single events were measured. Data was analysed using FlowJo 9.6.4 software (Treestar).

### Fluorescence-based cell imaging

Bright-field images of primary fibroblasts, melanocytes and UACC-62 melanoma cells were acquired 6 days after infection with lentiviruses coding for control or CPEB4-shRNA, using a Nikon ECLIPSE TiE fluorescence microscope (Izasa). For visualization of mitotic cells *in vitro*, control and shCPEB4-expresing UACC-62 cells were fixed with 4% paraformaldehyde and stained with α-Tubulin (dilution 1:500) and DAPI, for subsequent imaging with a 20x HCX PL APO 0.7 N.A. oil-immersion objective using a TCS-SP5 (AOBS-UV) confocal microscope (Leica Microsystems). For time-lapse videomicroscopy of cell cycle progression, SK-Mel-103 melanoma cells were transduced with RFP-Cdt1 and GFP-Geminin fusion proteins (FUCCI system) as previously reported^41^. Control or CPEB4 shRNA infected cells were plated at day 10 post-transduction in eight-well glass bottom plates, and both fluorescence and bright-field images were captured every 10 min during 20 h using 20x UPlan Sapo 0.75 N.A. dry objective on a Delta Vision RT microscope (Applied Precision) coupled to a CO_2_ and temperature-controlled incubation chamber. See additional detail on the imaging systems used in the Supplementary Methods.

### Histological analyses

Tissue sections were deparaffinized, incubated overnight with primary antibodies at 4 °C in a humidified chamber and then rinsed and incubated with fluorescent secondary antibodies for 1 h at room temperature. For immunohistochemical visualization and quantification of CPEB4 and RAB27A expression, primary antibodies used were CPEB4 mouse monoclonal clone ERE93C (dilution 1:50) and RAB27A rabbit antibody HPA001333 (Sigma; dilution 1:50). Secondary antibodies were anti-mouse Alexa Fluor 555 and anti-rabbit Alexa Fluor 488 (Life Technologies; dilution 1:400) and DNA was counterstained with DAPI. Negative controls were obtained by omitting the primary antibody. Image mosaics were acquired at 20 × HCX PL APO 0.7 N.A. dry objective using a TCS-SP5 (AOBS-UV) confocal microscope and were processed with the ‘intelligent matrix screening remote control' tool essentially as previously described^31^. Images were subsequently analysed with Definiens XD software to determine CPEB4 and RAB27A cytoplasmic intensities per cell. Signal intensities were quantified in maximum range of RGB colour scale (0–255). Data points were pseudo-coloured based on red/green signal intensity ratio (red, ratio>2; green, ratio<0.5; yellow, ratio 0.5–2). Signals below 10% of maximum pixel intensity (25/255) were considered as background and not considered for ratio calculations. See Supplementary Methods for additional detail on antibodies and quantification procedures.

### RNA immunoprecipitation (RIP)

For RNA immunoprecipitation, SK-Mel-103 cells were either left non-infected or were transduced with lentiviruses coding for CPEB4 shRNA. 6 days after infection cells were fixed at 80% confluence in 1% formaldehyde for 10 min at room temperature (RT) and were lysed in RIPA buffer containing protease and RNase inhibitors (Applied Biosystems) essentially as described before^62^. Lysates were sonicated in a Bioruptor Standard (Diagenode) for 10 min at low intensity and then precleared. Samples were immunoprecipitated using anti-CPEB4 antibody (ab830009, abcam) or rabbit IgG (Sigma) coupled to Dynabeads Protein A (Invitrogen) for 3 h at 4 °C. RNA elution was performed by two consecutive incubations at 55 °C for 30 min and at 65 °C for 45 min in RIPA buffer containing 50 μg proteinase K (Roche Applied Science), 1% SDS, 200 mM NaCl and 10 mM EDTA. Supernatants were digested with DNase I for 10 min at RT and RNA was extracted with TRI Reagent (Sigma) following manufacturer's protocol. For RNA sequencing, samples were processed by the CNIO Genomics Unit as described in the following section. For CPEB4 target validation, three additional independent RIP experiments were performed in SK-Mel-103 and UACC-62 cells, non-infected or transduced with CPEB4 shRNA. All immunoprecipitated RNA and 1 μg of RNA extracted from inputs were retrotranscribed using SuperScript III Reverse Transcriptase (Invitrogen) and subjected to quantitative-PCR. Primers for quantitative RT-PCR validation of candidate genes are described in the Supplementary Materials. Fold enrichment of target mRNAs in the immunoprecipitated fraction was calculated after normalization with the gene expression from the inputs.

### RNA sequencing and bioinformatic analyses

After RNA immunoprecipitation RNA integrity was evaluated by Agilent 2100 bioanalyzer using RNA 6000 Pico kit following manufacturer's recommendations. Twenty nanograms of RNA per sample were processed with Ribo-Zero Gold Kit (Epicentre, Cat. No. RZHM11106/RZG1224) for ribosomal RNAs removal. cDNA libraries were generated and sequenced on Illumina Genome Analyzer II × with SBS TruSeq v5 reagents following manufacturer's protocols.

Fastq files^63^ with 40-nt single-end sequenced reads obtained from two independent RIP-seq experiments were quality-checked with FastQC (Andrews, http://www.bioinformatics.babraham.ac.uk/projects/fastqc/) and aligned to the RefSeq human genome annotation data set GRCh37/hg19 (UCSC) with TopHat-2.0.4 (ref. 42) (using Bowtie 0.12.7 (ref. 64)) and processed with Samtools 0.1.16 (ref. 65), allowing two mismatches. For each read, the alignment with the best score was considered. An additional alignment was performed allowing 20 multihits for each read (TopHat default value). Assembly of transcripts, estimation of their abundances and differential expression were calculated with Cufflinks 1.3.0 (ref. 42). EdgeR software^66^ was used for an alternative analysis of the differential expression of the transcripts. The transcripts with FPKM expression values lower than 0.05 in both conditions were excluded of the analysis. Differences were considered significant when FDR-adjusted *P* values were lower than 0.05. Noncoding RNAs were excluded for the final selection of candidates.

The CPEB4-bound transcripts overlapping in RIP-seq replicates were then mined by GSEA across CCLE data sets, which encompasses 61 cell lines from melanoma, 44 from pancreatic cancer, and over 900 examples of 37 tumour types. Genes were ranked based on t statistic value. After Kolmogorov–Smirnoff testing, those gene sets showing FDR <0.25, a well-established cutoff for the identification of biologically relevant gene sets^67^, were considered enriched between classes under comparison.

CPEB4 targets identified by RIP-Seq in SK-Mel-103 melanoma (this study) and RWP-1 pancreatic cell lines^35^ were analysed for significantly enriched (*P*≤0.05) GO biological processes (database 02.10.2015) by using Cytoscape^68^ v3.2.1 and the ClueGO plug-in^69^ v2.1.7. For SK-Mel-103 panel, sizes of the nodes reversely represent the statistical significance of the terms (*P-*values were subjected to Bonferroni step down correction for multiple testing). For RWP1 pancreatic cells enriched GO terms were combined into hubs (Supplementary Fig. 6a; represented as circles, whose diameter is proportional to the number of gene clusters per hub). Pearson and Spearman correlation tests were performed to define the correlation in RIP-seq targets identified in melanoma and pancreatic cancer cells (*P* and *r* coefficient values >0.5 were considered significant).

CPEB4-bound transcripts in melanoma cells were further analysed to predict their regulation by cytoplasmic polyadenylation. A previously validated prediction algorithm^43^ was used to define the translational behaviour of the mRNAs. Briefly, this algorithm screens 3′-UTR regions for the presence of cytoplasmic polyadenylation-associated factors binding sites such as the cytoplasmic polyadenylation element (CPE), the polyadenylation hexanucleotide (HEX), AU-rich elements (ARE) and the Pumilio binding site (PBS). The specific elements and sequences considered are depicted in Fig. 7. In a second round, the software analyzes the distribution and combination of the elements to identify ‘activation' and ‘repression' motifs in candidate 3′-UTRs. mRNAs predicted to be regulated by cytoplasmic polyadenylation in melanoma were further analysed using IPA tool (http://www.ingenuity.com/products) and GSEA.

### Polyadenylation assays

RNA ligation-coupled RT-PCR was performed as described^70^ with some modifications. 4 μg of total RNA extracted from SK-Mel-103, UACC-62 or RWP1 cells transduced with control or CPEB4 shRNAs were ligated to 0.4 μg SP2 anchor primer (P-5′-GGTCACCTCTGATCTGGAAGCGAC-3′-NH2) using T4 RNA ligase (New England Biolabs). This was followed by retrotranscription with ProtoScript II reverse transcriptase (New England Biolabs) using 0.4 μg of ASP2T reverse-anchor primer (GTCGCTTCCAGATCAGAGGTGACCTTTTT), according to manufacturerś protocol. cDNA samples were then digested with 2 μg RNase A (Qiagen) and amplified by PCR using Platinum Taq polymerase (Invitrogen), ASP2T reverse-anchor primer and a specific forward primer for each mRNA analysed, following manufacturer's instructions. PCR products were resolved in 2% agarose gels for visualization. Poly(A) tail digestion by RNase H (New England Biolabs) before RNA ligation in the same initial RNA samples were used as control of the PCR reaction specificity. The primers used for polyadenylation assays are shown in Supplementary Methods.

### Animal experiments

Mouse xenograft models were generated by subcutaneous implantation of SK-Mel-28 (2.5 × 10^6^), SK-Mel-103 (1.0 × 10^6^) or UACC-62 (5 × 10^6^) in 8 week-old athymic nude (Crl:NU(NCr)-*Foxn1*^*nu*^) female mice (Charles Rivers). To this end, cells were harvested at day 7 after transduction with control or CPEB4 shRNAs, resuspended in 0.1 ml PBS (SK-Mel-103 cells) or matrigel (BD Bioscience) at 1:3 ratio in PBS (SK-Mel-28 and UACC-62 cells) and injected in the back of the animals (*N*=5 per group). Tumour growth was measured blinded to the experimental conditions at the indicated time intervals. Tumour volume was estimated using a caliper and calculated as V=axb^2^ × 0.52, where ‘a' stand for the bigger and ‘b' for the smaller diameter of the tumour. When control tumours exceeded 1 cm^3^ size mice were sacrificed and xenografts were surgically excised and processed for histopathology. All experiments with mice met the Animal Welfare guidelines and were performed in accordance with protocols approved by the Ethics Committee of the Instituto de Salud Carlos III.

### Statistical analyses

Multi-tumour CPEB4 mRNA expression box plot was extracted by the CCLE database (http://www.broadinstitute.org/ccle/home)^37^ using cBioPortal. Additional cross-tumour profiles in tumour specimens and the corresponding enrichments in melanoma were extracted from Oncomine from the data sets indicated in corresponding figure legends. Differential CPEB4 expression among benign and malignant human melanocytic lesions was evaluated by χ^2^-test. Cell proliferation and tumour growth curves were analysed by two-way analysis of variance (mixed model) considering matching among the measures at different time points. In all the cases *P*<0.05 was considered significant. When indicated, *P* values were represented as follows: ‘***' for *P*<0.001, ‘**' for *P*<0.01, ‘*' for *P*<0.05 and ‘NS' for not significant. CPEB4 protein expression was compared between melanoma and non-melanoma tumour cell lines using two-tailed unpaired Student's *t*-test. RAB27A and MITF differential expression found in murine-xenografted tumours were analysed by two-tailed unpaired Student's '*t*-test. CPEB4 and RAB27A coexpression in human melanoma specimens was evaluated by Pearson test (with *P*>0.5 was considered significant). For GSEA, gene sets showing FDR<0.25 after Kolmogorov-Smirnoff testing were considered enriched between classes under comparison.

### Data availability

RIP-seq data has been deposited in NCBI's Gene Expression Omnibus (GEO Series accession number GSE75773). All additional relevant data and experimental detail, including the bioinformatic tools for CPE identification are available from the authors upon request.

## Additional information

**How to cite this article:** Pérez-Guijarro, E. *et al*. Lineage-specific roles of the cytoplasmic polyadenylation factor CPEB4 in the regulation of melanoma drivers. *Nat. Commun.* **7,** 13418 doi: 10.1038/ncomms13418 (2016).

**Publisher's note:** Springer Nature remains neutral with regard to jurisdictional claims in published maps and institutional affiliations.

## Supplementary Material

Supplementary Information Supplementary Figures 1 - 11, Supplementary Methods and Supplementary References

Supplementary Movie 1 Time lapse imaging of cell cycle progression in CPEB4 expressing melanoma cell lines (SK-Mel-103). Images correspond to SK-Mel-103 labeled with RFP-Cdt1 and GFP-Geminin fusion proteins (FUCCI system)4, and transduced with shRNA control. Video was captured for 19 h at 10 min intervals in a Leica DMI6000 B fluorescence microscope coupled to a CO2 and temperature-controlled incubation chamber. Note red-into-green conversion as the melanoma cells divide. Equivalent imaging of CPEB4-depleted cells can be found in Supplementary Movie 2.

Supplementary Movie 2 Time-lapse imaging for the visualization of cell cycle defects accumulated in CPEB4 depleted melanoma cells. 19 hours time-lapse video of SK-Mel-103 melanoma cells expressing RFP-Cdt1 and GFP-Geminin fusion proteins upon transduction of CPEB4 shRNA (sh1). Images were captured at 10 min intervals. In contrast to the red-into-green conversion found for control shRNA cells (Supplementary Movie 1), CPEB4-depleted cells arrested in G1 as manifested by the accumulation of red-fluorescent cells. CPEB4-deficient cells that can progress into cell cycle (green fluorescence) displayed obvious mitotic defects in spindle formation.

Supplementary Data 1 RIP-Seq analysis for the identification of CPEB4-bound transcripts in melanoma. RNA collected from control or shCPEB4 SK-Mel-103 cells (two independent replicates each) was subjected to crosslinking and immunoprecipitation followed by Ribo-Zero treatment for elimination of ribosomal RNA. Reads were aligned to the human genome (GRCh37/hg19) with TopHat-2.0.4. The best hits were analyzed with Cufflinks to identify transcripts not expressed or downregulated I the shCPEB4 conditions (i.e. enriched in the controls). Tab A and B correspond to replicates 1 and 2 respectively. Transcripts with q-values<0.05 (FDR adjusted p-values) were considered significant. FPKM: Fragments per kilobase of transcript per million mapped reads.

Supplementary Data 2 Network analysis of functional categories enriched in CPEB-bound targets identified by RIP-Seq in melanoma cells. GO-terms identified as significantly enriched in SK-Mel-103 transduced with shControl vs shCPEB4 (GSEA, p<0.05). Gene clusters are numbered as in Fig. 6c. Indicated are also GO accession numbers, the corresponding p values and the identity of the CPEB4 targets in each gene category.

Supplementary Data 3 Network analysis of functional categories enriched CPEB-bound targets identified by RIP-Seq in pancreatic cells. Significantly enriched GO-functional categories (GSEA p<0.05) in gene lists extracted from RIP-Seq analyses performed in RWP1 pancreatic cancer cells. Gene clusters are numbered as in Supplementary Fig. 6. GO accession numbers, the corresponding p values and the identity of the CPEB4 targets in each gene category are also indicated. Note the minimum overlap with genes in Supplementary Data 2 (melanoma SK-Mel-103).

Supplementary Data 4 CPE-containing transcripts identified as CPEB4 targets by RIP-Seq in SK-Mel-103 melanoma cells. (Tab A) Genes with bona fide CPE sites in 3'UTR and directly recognized by CPEB4 as defined by RIP-Seq. The Top 10 functionally-enriched gene functions identified by iPA for these genes are listed and described in Tab B. (Tab C) iPA-identified enriched functions grouped by categories. (Tab D) all gene clusters identified by iPA as significantly enriched in the CPE-expressing genes bound to CPEB4 in SK-Mel-103 melanoma cells, with the corresponding GO accession numbers, p values and gene names.
