# Supplementary material for: Lineage-specific roles of the cytoplasmic polyadenylation factor CPEB4 in the regulation of melanoma drivers
Source: Nat Commun. 2016 Nov 18;7:13418. doi: 10.1038/ncomms13418 (PMC5120223; doi:10.1038/ncomms13418)
Supplement: Supplementary Information — Supplementary Figures 1 - 11, Supplementary Methods and Supplementary References [file ncomms13418-s1.pdf]

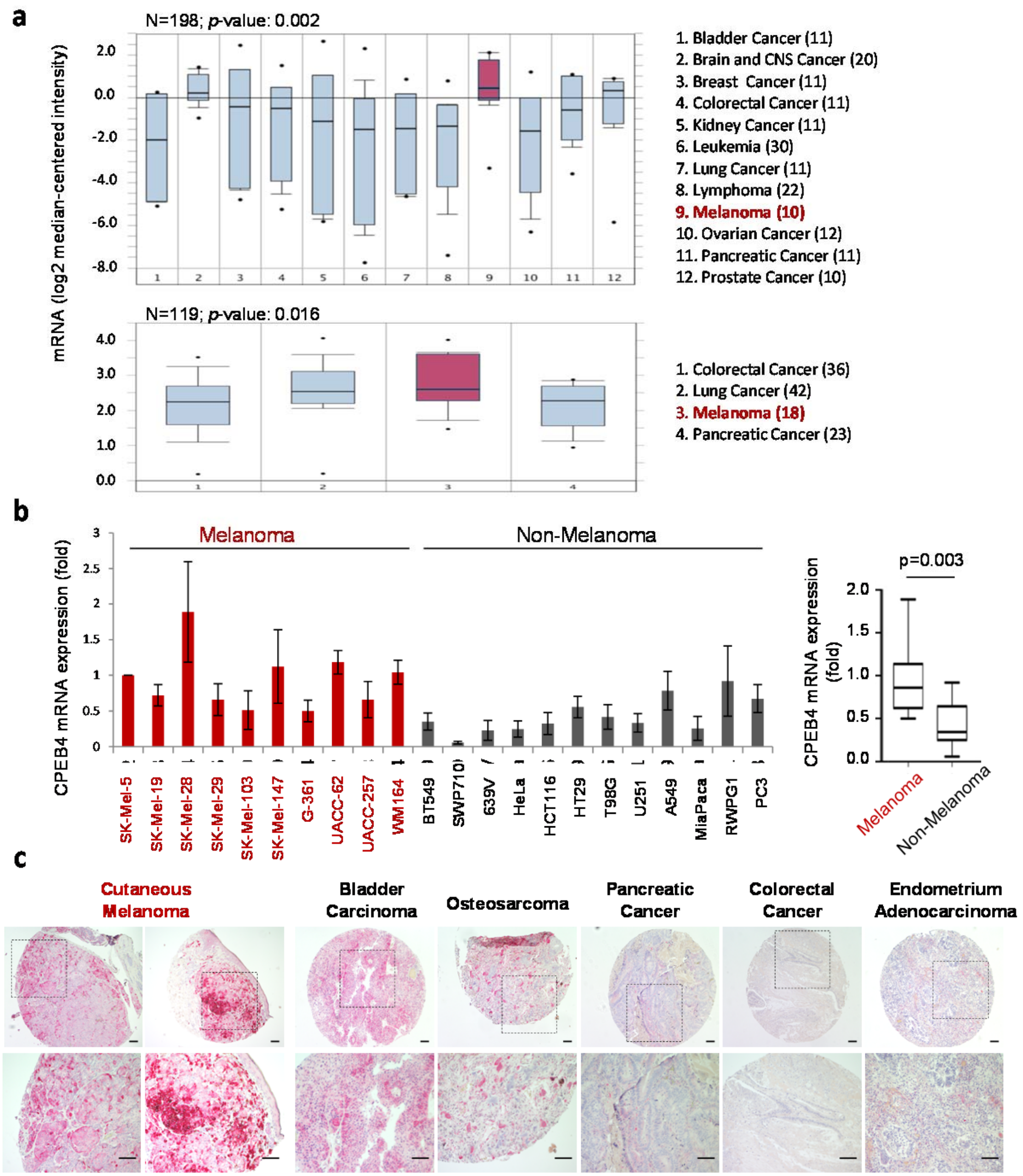

Supplementary Figure 1. CPEB4 expression in melanoma versus non-melanoma tumors. (a) Relative *CPEB4* mRNA expression across the indicated cancer types as extracted by Oncomine from Ramaswamy Multi-cancer[1] (upper panel; shown 170 out of the 198 cell lines of this dataset), and Wagner Cell Line set[2] (bottom panel; N=119). The number of cell lines from each tumor type is indicated in parenthesis. p values for melanoma-enriched CPEB4 are also indicated for each dataset. **(b)** *CPEB4* mRNA levels analyzed by RT-qPCR in the indicated melanoma (red) and non-melanoma (black) cell lines. Data is represented as means ± SEMs of three experiments in triplicates. Aggregate levels for the melanoma vs. non melanoma cases analyzed are depicted in the right graph. p: Student's t-test p-value. **(c)** Representative examples of CPEB4 expression detected by immunohistochemistry in tissue microarrays (TMAs). Nuclei are counterstained with hematoxilin. The number of samples analyzed per tumor type is indicated in Supplementary Methods. Scale bars represent 50 µm.

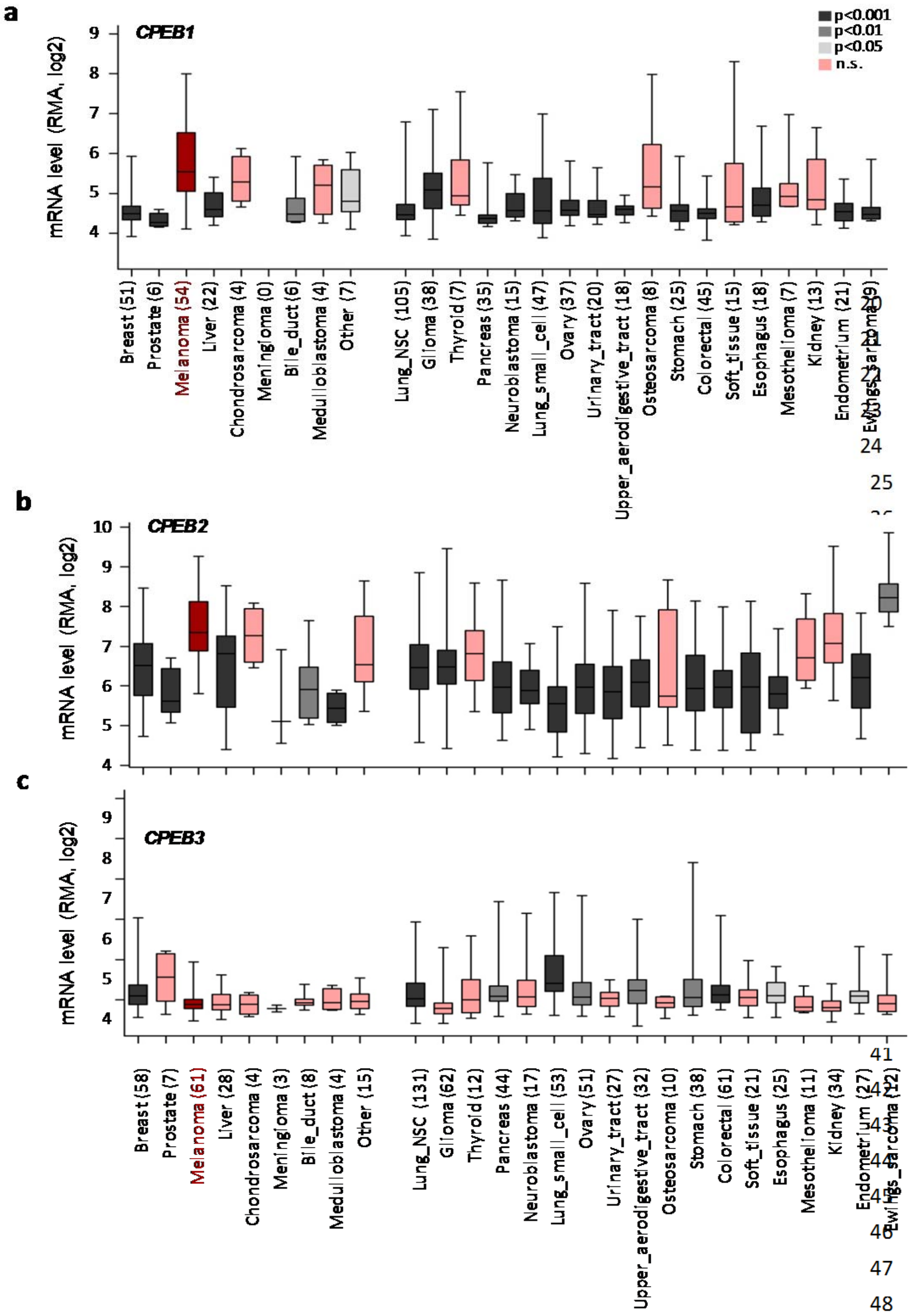

Supplementary Figure 2. mRNA expression of *CPEB1-3* in melanoma and non-melanoma tumor cell lines from CCLE dataset. *CPEB1* **(a)***, CPEB2* **(b)** *and CPEB3* **(c)** mRNA expression in 27 solid tumor types including melanoma extracted from the CCLE database (CCLE_Expression_Entrez_2012-10-18.res). The different tumors are listed following the relative expression of CPEB4 as defined in Fig. 1a. The number of cell lines from each cancer type is indicated in parenthesis. Box colors represent the p-values from pairwise comparisons between melanoma and each of the indicated tumor types.

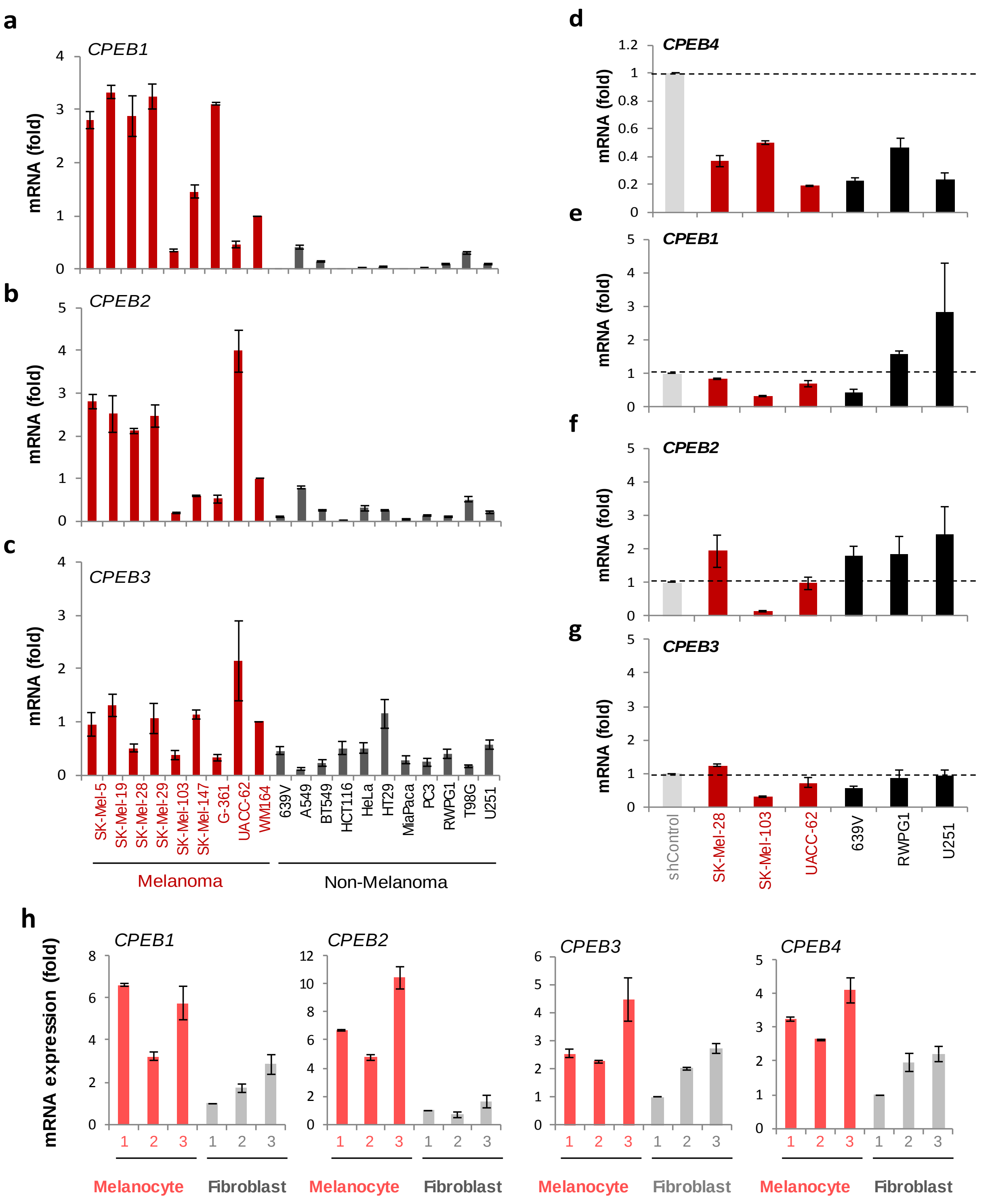

Supplementary Figure 3. mRNA expression of CPEB family members in melanocytic and non-melanocytic cells. (a-c) mRNA expression of *CPEB1-3* determined by quantitative qRT-PCR in the indicated melanoma and non-melanoma tumor cell lines. **(d)** CPEB4 downregulation measured by quantitative qRT-PCR upon transduction of CPEB4 shRNA in the indicated cell lines. *CPEB4* mRNA expression in shControl transduced cells is used as reference. Levels of *CPEB1, CPEB2* and *CPEB3* mRNA in these CPEB4-depleted cells are depicted in panels **(e-g)**, respectively. **(h)** Relative mRNA levels of CPEB1-4 defined by qRT-PCR in primary melanocytes and genetically-matched primary fibroblast. Data are shown as means ± SEMs of two experiments in triplicate.

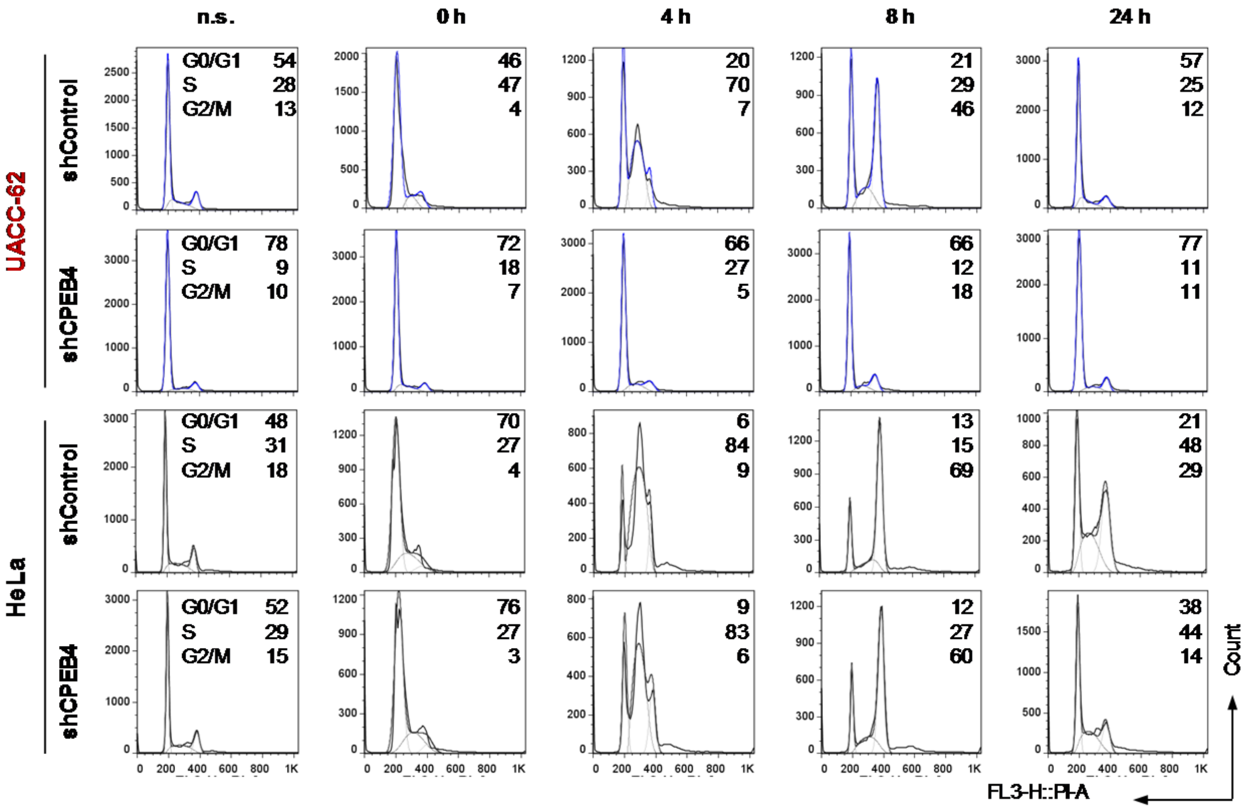

Supplementary Figure 4. CPEB4 depletion compromises cell cycle proliferation in melanoma cells. Time-course analysis of the ability of the indicated cell populations to progress through the cell cycle after release from thymidine block. The percentages of cells at the G0/G1, S or G2/M phases were determined by propidium iodide (PI) staining and calculated using FlowJo software. n.s.: non-synchronized.

a

Sample	Total read number		Reads aligned (used by Cufflinks)	
	Replicate 1	Replicate 2	Replicate 1	Replicate 2
RIP-Seq basal conditions	21507059	18246637	17872204	15491868
RIP-Seq after shCPEB4	16403772	17580976	12953866	14400648

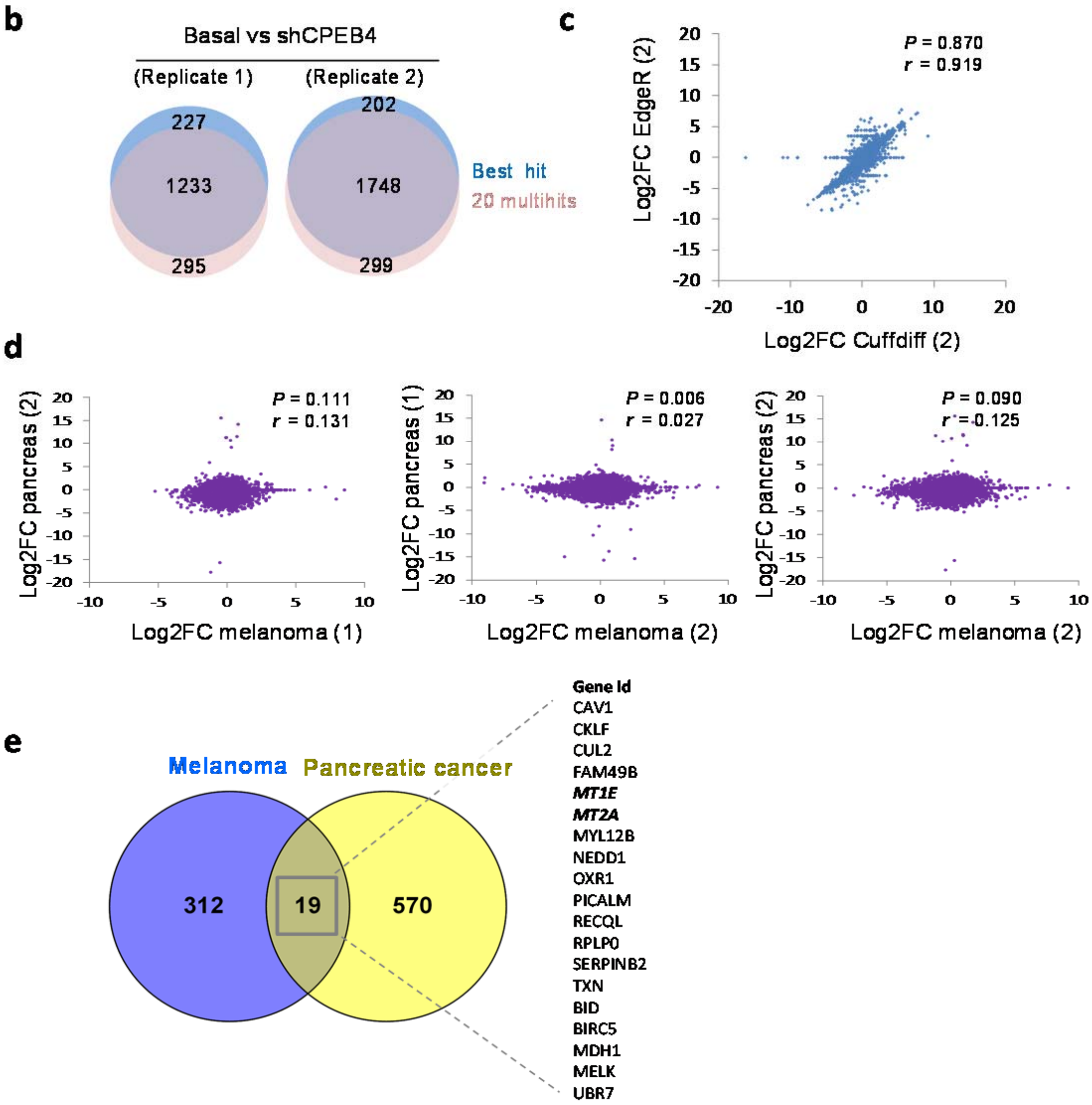

Supplementary Figure 5. High correlation of RIP-seq data using different methodologies for analysis. (a) Total read counts obtained by Illumina sequencing and number of reads aligned by TopHat-2.0.4 software and used for differential expression analysis by Cuffdiff from two RIP experiments in SK-Mel-103 cells. **(b)** Venn diagramas comparing results obtained by TopHat-2.0.4 alignment using the best match score (best hit) or allowing for 20 multihits. Data are showed as the number of immunoprecipited transcripts found differentially expressed in control- versus shCPEB4-transduced cells. **(c)** Correlation of fold change expression (Log2 scale, Log2FC) of immunoprecipited transcripts identified by Cuffdiff or EdgeR in an independent replicate of data in Fig. 5a. **(d)** Comparison of CPEB4-bound mRNAs (Log2FC) in melanoma cells vs RWP-1 pancreatic cancer cell line (the latter extracted from Ref[3]). Graphs show results for the indicated replicates of each cell line. **(e)** Venn diagrams comparing CPEB4-bound mRNAs in SK-Mel-103 melanoma cells with respect to previous reports for RWP1 pancreatic cells. Pearson coefficient (P) and Spearman rank correlation coefficient (r) values are indicated in the corresponding panels.

a

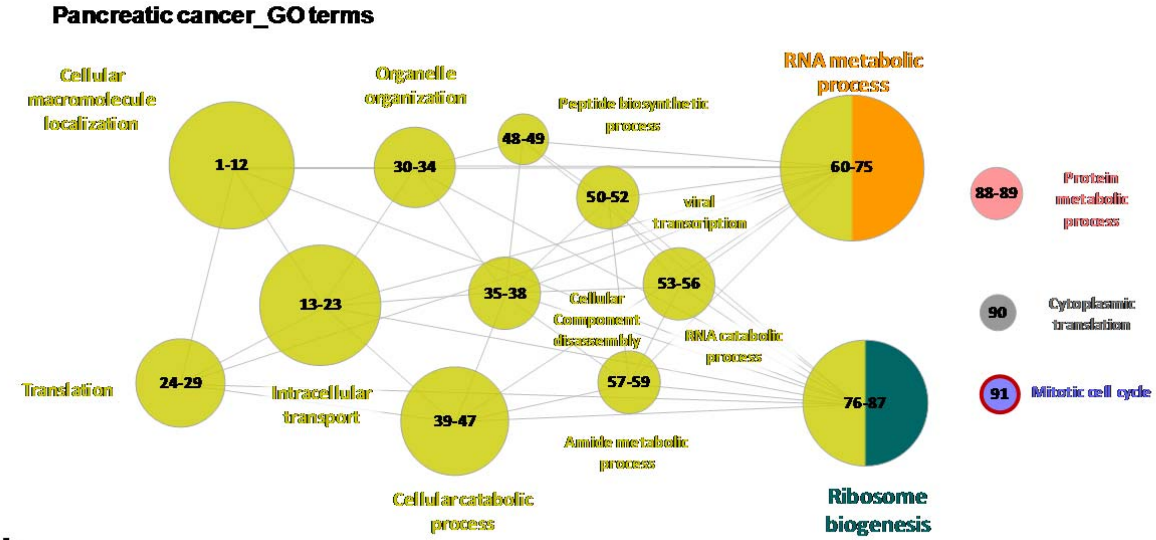

b

IPA functional category (Melanoma)	p-Value	-Log (p-value)	# genes	Genes
Cell Cycle	2.75E-12	11.56	45	ANLN,APP,ARNTL2,BUB1,BUB1B,CAV1,CD44,CDCA8,CDK1,CDK6,CENPE,CKAP2,CKS1B,CUL2,DYNLT3,EIF4G2,FBXO5,FKBP1A,FOSL1,GMNN,HHEX,HMGB1,KIF2A,MAD2L1,MCM2,NCOA4,NEDD1,NPM1,NUF2,NUSAP1,OSBPL9,PLAG1,PLK4,PRKAR2B,PTMA,PTPN2,RCC1,RELA,S1PR1,SKA3,TMPO,TOP2A,TXN,VEGFA,ZWINT,RAB27A, DEK
Cellular Growth and Proliferation	9.75E-09	8.01	90	ABL2,ACP1,APP,ARMC10,ARNTL2,ASPH,ATF2,B4GALT6,BCAT1,BIRC3,BUB1,BUB1B,CAV1,CD44,CDCA2,CDCA7,CDCA7L,CDCA8,CDK1,CDK6,CKLF,CKS1B,CPSF4,CTNND1,CTSB,CUL2,DIAPH1,DUSP5,EIF4G2,ETS2,ETV5,EXOSC9,FBXO9,FKBP1A,FKBP5,FOSL1,FOXP1,FUS,GMNN,H2AFZ,HDGF,HHEX,HMGB1,HNRNPC,HNRNPF,ILF3,LDHA,MAD2L1,MANF,MBNL1,MCFD2,MCM2,MT1E,MT2A,MTF2,MYBL1,NCOA4,NEIL3,NPM1,NRP2,NT5C3A,ODC1,PEX2,PLAG1,PLK4,PRKAR2B,PTMA,PTPN2,PTPN22,RAB27A,RECQL,RELA,RFC1,RNF7,S1PR1,SERPINB2,SET,SHMT2,SLC7A11,SLC7A5,SSX2IP,TIMP3,TIPIN,TMEM2,TMPO,TOP2A,TXN,VEGFA,WHSC1,WWTR1
Cell Death and Survival	2.15E-08	7.67	72	ABL2,ALDH1A3,API5,APP,ARMC10,ATF2,ATG16L1,BIRC3,BUB1,CASP8AP2,CAV1,CBX5,CD44,CDCA2,CDK1,CDK6,CKAP2,CTSB,CUL2,DUT,EIF4G2,ETS2,ETV5,EXO1,FAM72A,FBXL5,FBXO5,FKBP1A,FKBP5,FOSL1,FOXP1,FUS,GMNN,HDGF,HMGB1,HNRNPC,HSPE1,ILF3,LDHA,MAD2L1,MCM2,MME,MT1E,MT2A,MYBL1,NCOA4,NPM1,NUSAP1,ODC1,OPA1,PCBP2,PHF17,PLK4,PRKAR2B,PTMA,PTPN2,PTPN22,RAB27A,RELA,RFC1,RNF7,RPLP0,S1PR1,SERBP1,SERPINB2,SET,SH3GLB1,TIMP3,TOP2A,TXN,VEGFA,YWHAZ
Gene Expression	2.18E-08	7.66	55	APP,ARNTL2,ATF2,C1D,CAV1,CBX5,CD44,CDK1,CKAP2,DEK,DUSP5,EIF4G2,ELAVL2,ETS2,ETV5,FKBP1A,FOSL1,FOXP1,GMNN,H1F0,HDGF,HHEX,HMGB1,HNRNPC,ILF3,LRP8,MATR3,MME,MORF4L2,MYBL1,NCOA4,NPAS2,NPM1,PEX2,PICALM,PLAG1,POLR3G,PRKAR2B,PSMC3IP,PTMA,RELA,RFC1,S1PR1,SET,TIMP3,TMPO,TOP2A,TRIP13,TROVE2,TXN,VEGFA,WHSC1,WWTR1,YWHAZ,ZNF367
Cellular Assembly and Organization	1.11E-07	6.95	10	BUB1,CENPE,EXO1,MAD2L1,NUF2,NUSAP1,RCC1,SKA3,TOP2A,ZWINT
Cellular Development	4.35E-07	6.36	46	ABL2,APP,ATF2,BUB1,CAV1,CD44,CDCA2,CDCA8,CDK1,CDK6,CKS1B,CTNND1,CTSB,DUSP5,ETS2,FBXO9,FKBP5,FOSL1,FOXP1,FUS,GMNN,H2AFZ,HDGF,HMGB1,LDHA,MCM2,MT2A,MYBL1,NPM1,NRP2,NT5C3A,ODC1,PRKAR2B,PTMA,PTPN22,RELA,RNF7,SERPINB2,SET,SLC7A11,TIMP3,TMEM2,TXN,VEGFA,WHSC1,WWTR1
Cancer	4.54E-07	6.34	90	ABL2,ALDH1A3,ANLN,ASF1B,ATF2,ATP5G3,BIRC3,BUB1B,BZW1,C8orf59,CASP8AP2,CAV1,CBX5,CD44,CDCA7,CDCA8,CENPE,CKLF,CKS1B,CTSB,DEK,DIAPH1,DUSP5,EIF4G2,ELAVL2,ETV5,EXO1,EXOSC9,FAM83D,FKBP1A,FKBP5,FUS,GMNN,GPR176,HDGF,HIST1H4A,HMGB1,HNRNPDL,HSPE1,KNSTRN,KRIT1,LDHA,LRP8,MAD2L1,MANF,MATR3,MCMBP,MME,MT1E,MT2A,MYBL1,NCOA4,NEIL3,NPAS2,NPM1,NUF2,NUSAP1,ODC1,ORC4,PLAG1,PTMA,RAD51AP1,RELA,RFC1,RIF1,RNF182,RPLP0,S100A2,S1PR1,SERBP1,SET,SLC38A1,SLC7A11,SLC7A5,SNX5,SPDL1,SSX2IP,THUMPD3,TIMP3,TM4SF1,TMPO,TMX2,TOP2A,TRIP13,TXN,TYW3,VEGFA,WHSC1,YWHAZ,ZWINT
Cellular Movement	9.77E-07	6.01	29	ABL2,APP,BCAT1,CAV1,CBX5,CD44,CTNND1,CTSB,DIAPH1,ETS2,ETV5,FKBP1A,FOSL1,FOXP1,HDGF,HHEX,HMGB1,NCOA4,NPM1,NRP2,ODC1,PICALM,PIP5K1A,RELA,SSX2IP,TIMP3,VEGFA,WHSC1,WWTR1
Metabolic Disease	2.54E-06	5.60	22	AKAP2,APP,CAV1,CDK1,CTSB,ETS2,HNRNPDL,LARP4,LRP8,MME,NAV3,OPA1,PICALM,PRKAR2B,RCC1,RELA,SET,TIMP3,TMEM2,TXN,WASF1,YWHAZ
Reproductive System Development and Function	3.19E-06	5.50	4	BUB1,BUB1B,MAD2L1,TRIP13

Supplementary Figure 6. Differential transcripts recognized by CPEB4 in melanoma *versus* pancreatic cancer cells. (a) Graphical representation of Gene Ontology biological processes (GO database 02.10.2015) enriched in the pancreatic cancer cell line RWP1, corresponding to RIP-Seq analyses extracted from Ref[3] and graphed with Cytoscape. The diameter of the circle reflects the number of GO terms in each of the functional categories. Additional information on the gene clusters (numbered from 1 to 91), and the corresponding CPEB4-bound targets per cluster are listed in Supplementary Data 3. **(b)** Summary of the functional processes with the highest enrichment in the SK-Mel-103 melanoma cells identified by IPA using the CPE-containing transcripts identified by RIP-seq as CPEB4 recognized transcripts; see Supplementary Data 4 for additional information. Genes validated by RNA Immunoprecipitation and qRT-PCR, as well as by PAT assays (i.e. to demonstrate direct binding and control of poly(A) tail length) are marked in red.

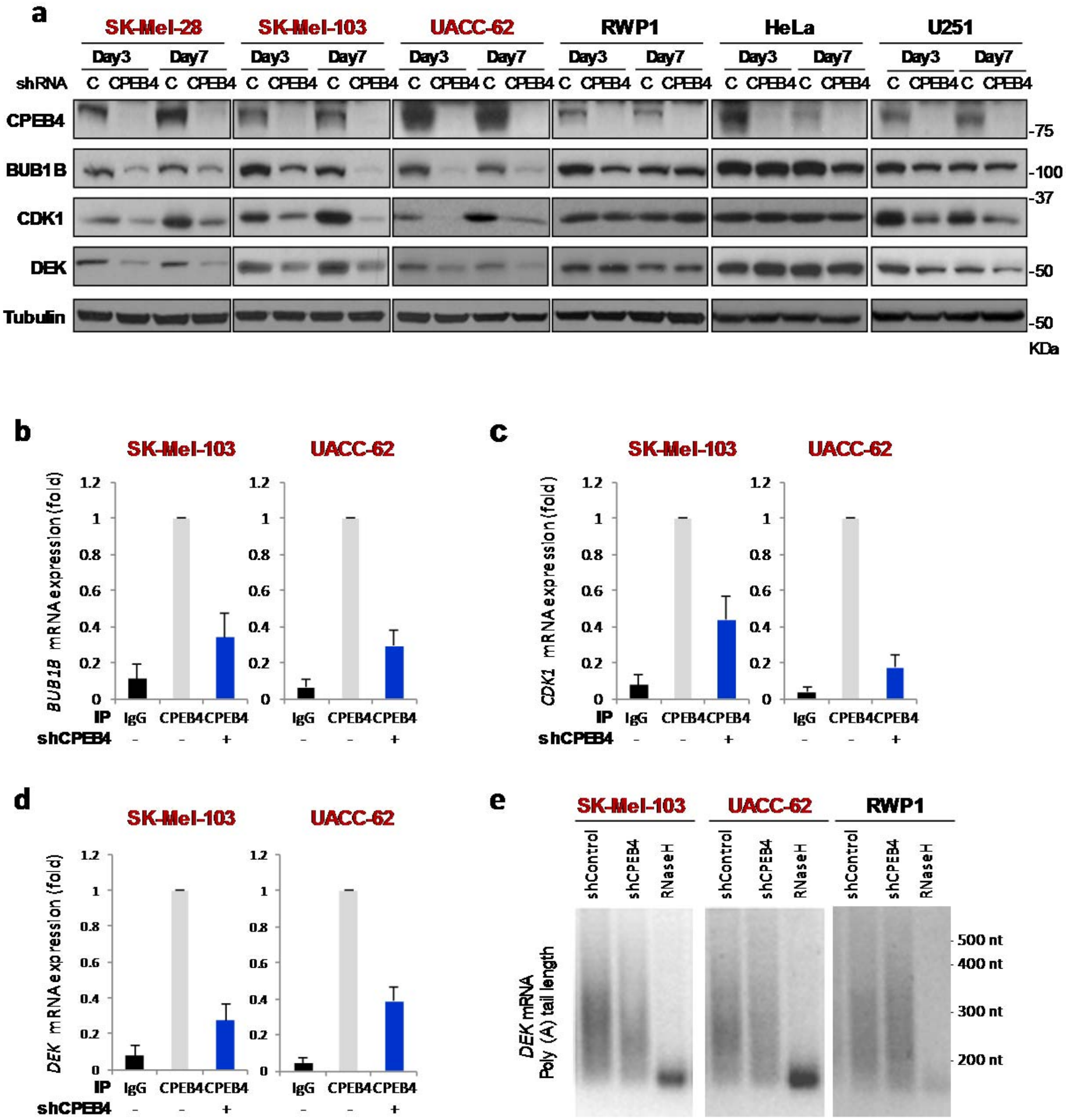

Supplementary Figure 7. Validation of novel pro-oncogenic signaling hubs as CPEB4 targets in melanoma. (a) Immunoblots showing BUB1B, CDK1 and DEK protein downregulation in melanoma (red) and non-melanoma (black) cell lines expressing CPEB4 shRNA(1). **(b-d)** *BUB1B* **(b)**, *CDK1* **(c)** and *DEK* **(d)** mRNA levels in the immunoprecipitated fraction of parental or shCPEB4 transduced cells. Data correspond to quantitative qRT-PCR obtained using RNA fractions crosslinked to CPEB4 antibody or rabbit IgG. Primers spanning the 3'-UTR regions of the indicated genes are listed in Supplementary Methods. mRNA levels were normalized against expression in the inputs (parental and shCPEB4-expressing cells) and data are presented as means ± SEMs from three independent RIP experiments. **(e)** Polyadenylation length test (PAT) of *DEK 3'UTR* in the indicated shControl or shCPEB4 transduced cell lines. RNase H was used for poly(A) tail removal to define the specificity of the amplification procedure[3]. nt: nucleotides

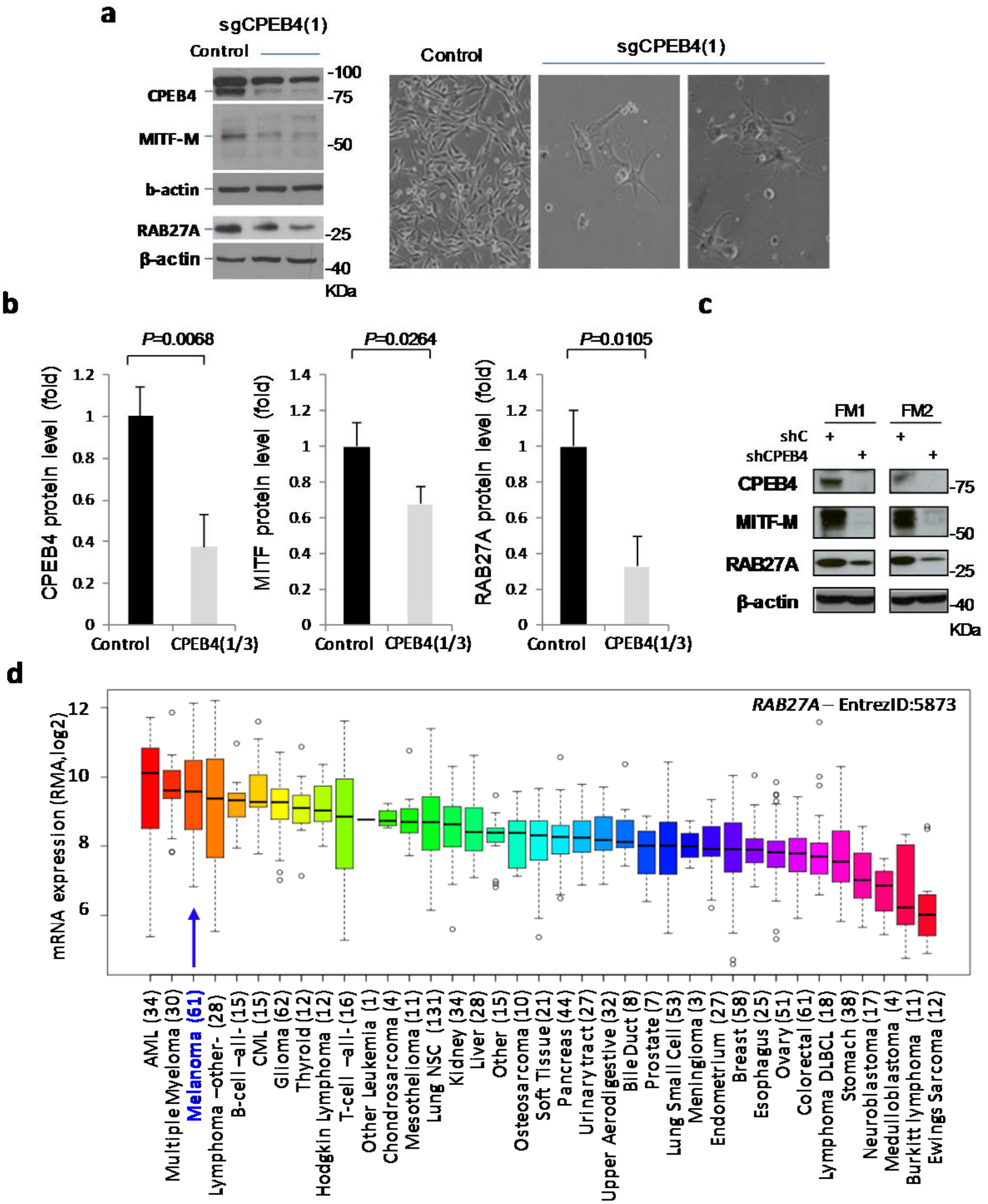

Supplementary Figure 8. Validation of *MITF* and *RAB27A* as a direct CPEB4 targets using CRISPR-Cas9 technology. **(a)** CPEB4 depletion by CRISPR-Cas9 genomic editing (gCPEB4-1) in UACC-62 cell line. MITF and RAB27A downregulation is also demonstrated by immunotblot (left panel). Representative images of cell clones showing the decrease in cell density by transfection of gCPEB4-1 are depicted in right panel. **(b)** Quantification of CPEB4, MITF and RAB27A protein levels upon CPEB4 depletion from immunoblots as showed in (a). Data is represented as means ± SEMs from three clones generated using 2 different CPEB4 guide RNAs (gCPEB4-1/3). **(c)** Downregulation of MITF and RAB27A in two independent preparations of foreskin melanocytes (FM1 and FM2) transduced with shCPEB4 and visualized by immunoblotting. **(d)** Box plots showing relative *RAB7A* mRNA levels across the different tumor types included in the CCLE dataset. The number of cell lines of each tumor type analyzed is indicated in parenthesis.

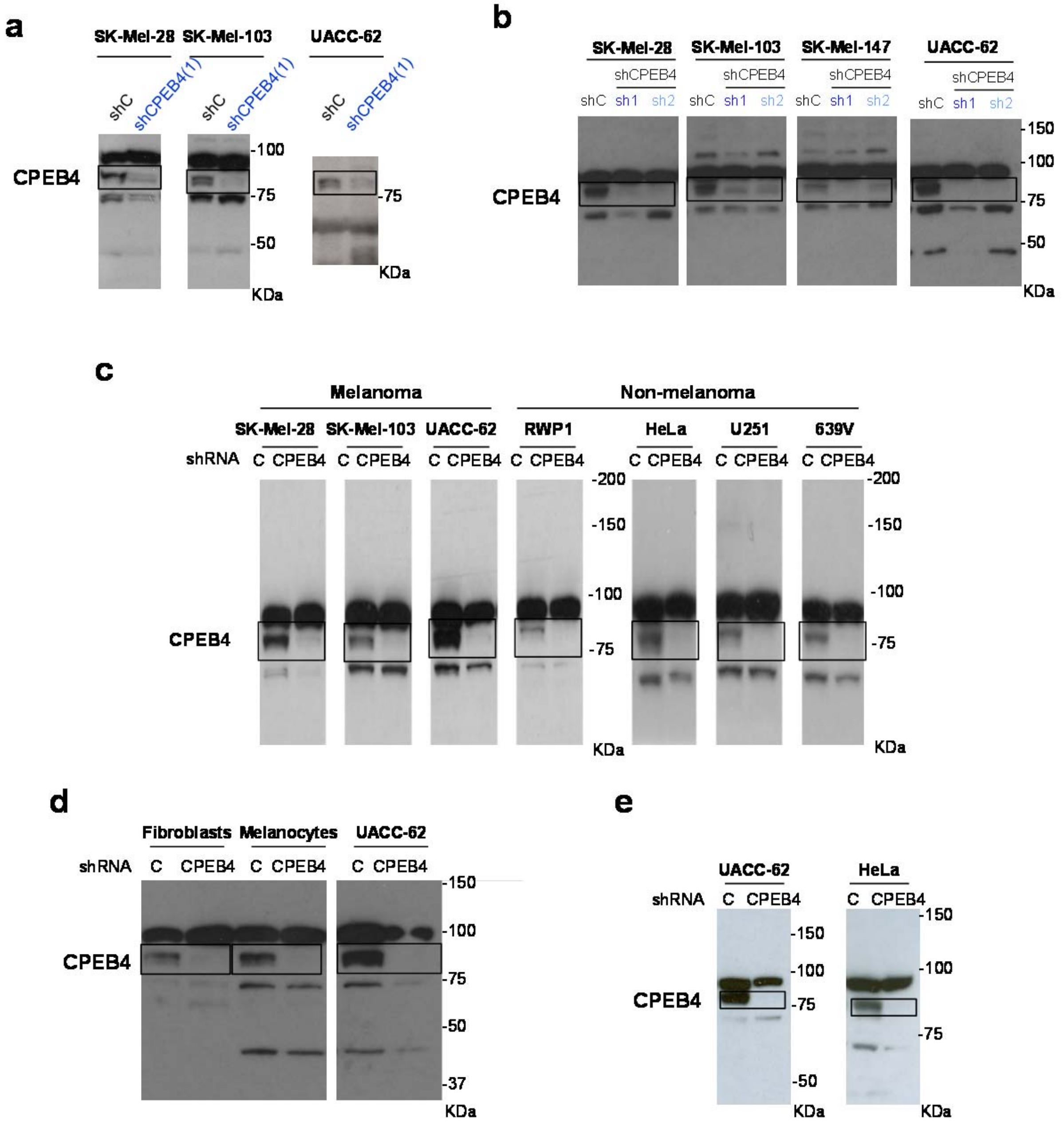

Supplementary Figure 9. Uncropped western blot images corresponding to Figure 2a (a), Figure 2c (b), Figure 3a (c), Figure 3c (d) and Figure 4e (e).

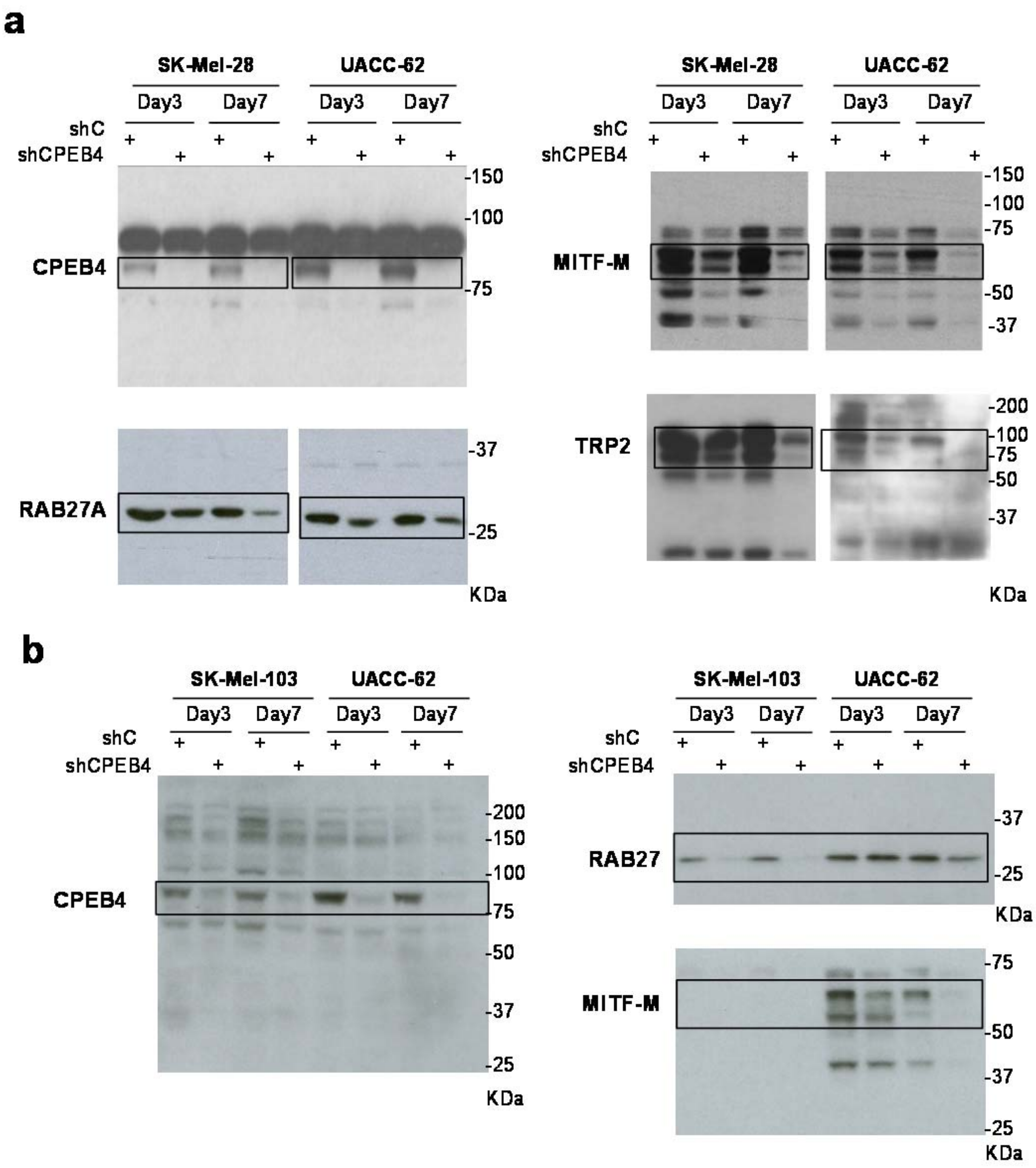

Supplementary Figure 10. Uncropped western blot images corresponding to Figure 8a (a) and Figure 9a (b).

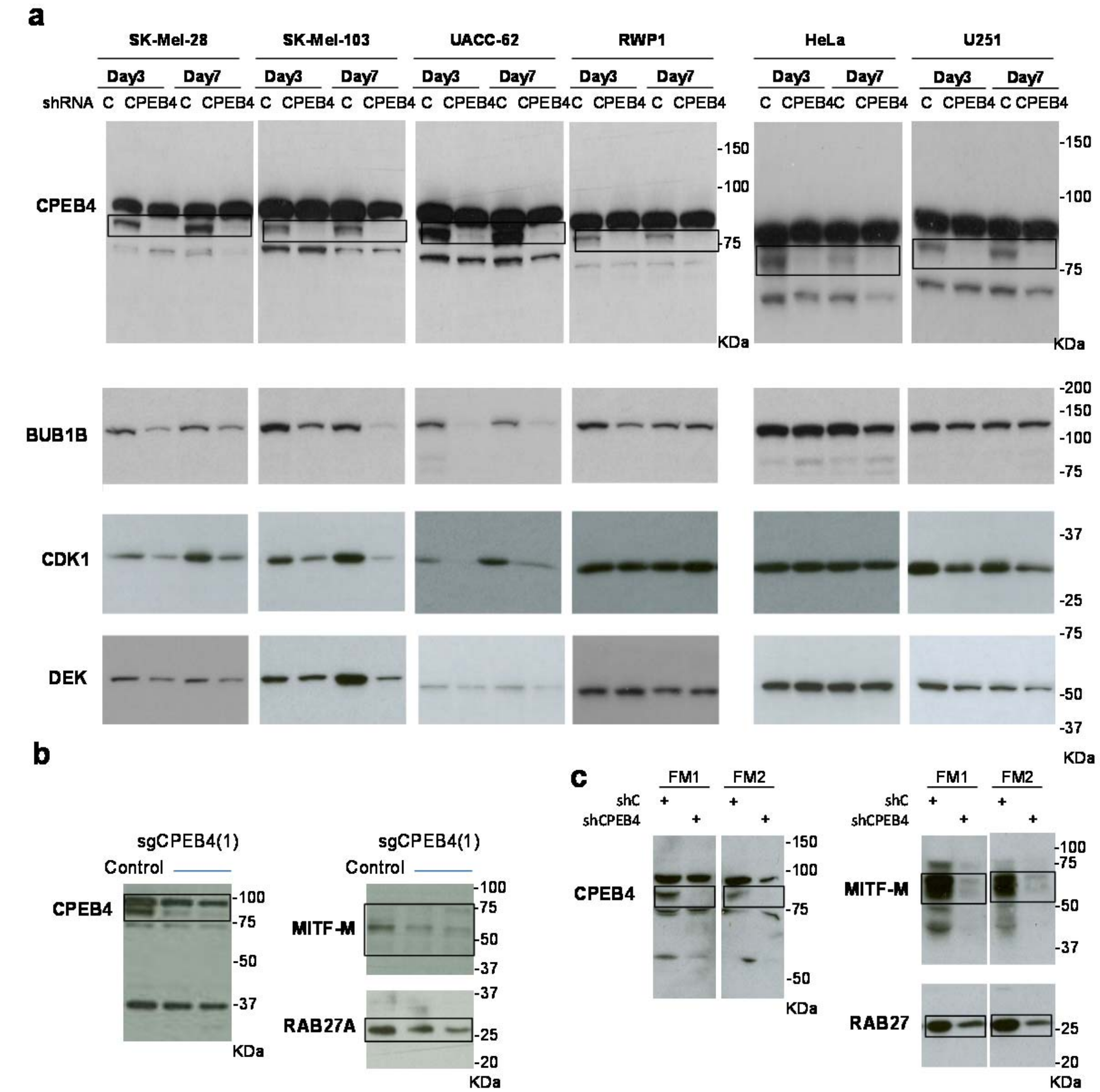

Supplementary Figure 11. Uncropped western blot images corresponding to Supplementary Figure 8a (b) Supplementary Figure 8a (b) and Supplementary Figure 8c (c).

Supplementary Methods

RNA extraction, RT-qPCR and Oligonucleotides

Total RNA was extracted and purified from cell pellets using RNeasy Mini-Kit (QIAGEN) following the manufacturer's instructions. 2 µg total RNA was reverse-transcribed into cDNA using the high capacity cDNA reverse transcriptase kit (Applied Biosystems), according to manufacturer´s protocol. 20 ng of the total cDNA were subjected to real-time polymerase chain reaction (qPCR) using Power SYBR® Green PCR Master Mix (Applied Biosystems). Assays were run in triplicates on the 7900HT Fast Real-Time PCR system (Applied Biosystems). HPRT was used as loading control to normalized mRNA expression. For quantitative RT-PCR the primers used were:

CPEB4 Fw: TGGGGATCAGCCTCTTCATA, CPEB4 Rv: CAATCCGCCTACAAACACCT
CPEB1 Fw: CCTGGGTATTAGCCGACAGT, CPEB1 Rv: GCCTCAGCATTTAGCATTCC
HPRT Fw: CCTGGCGTCGTGATTAGTGAT, HPRT Rv: AGACGTTCAGTCCTGTCCATAA

For RIP-qPCR the primers used were:

DEK Fw: GCCATGTTAAAGAGCATCTGTG, DEK Rv: CAGAAGGCTTTGGATGCATTA
BUB1B Fw: CTCGTGGCAATACAGCTTCA, BUB1B rv: CCAGGCTTTCTGGTGCTTAG
CDK1 Fw: AATGGAAACCAGGAAGCCTAGC, CDK1 Rv: GCCAGAAATTCGTTTGGCTGG
RAB27A Fw: GAAACTGGATAAGCCAGCTACAG, RAB27A Rv: ATATTTCTCTGCGAGTGCTATGG
MITF Fw: GCGCAAAAGAACTTGAAAAC, MITF Rv: CGTGGATGGAATAAGGGAAA

Primers used for polyadenylation assays were:

DEK: CTTGATAGTTACTCAGACACTAGGG
RAB27A: CATGATATAGTGCACACACAAAAGCCACC and MITF: GTCACCTGCTGTTGGATGCAGC

Gene silencing by CRISPR-Cas9 technology

CPEB4 gene were silenced by CRISPR-Cas9 technology. To this end, Zhang Lab platform (http://crispr.mit.edu/) was used to design guide sgRNA sequences targeting the first exon common to all CPEB4 isoforms. Oligonucleotides used were: CPEB4_01_Fw: CACCGAGCTGGGGCGAGCATACTTC; CPEB4_01_Rv: AAACGAAGTATGCTCGCCCCAGCTC; CPEB4_02_Fw: CACCGCCGTTATTAGCCGAAGCAGC; CPEB4_02_Rv: AAACGCTGCTTCGGCTAATAACGGC; CPEB4_03_Fw: CACCGCCGTTATTAGCCGAAGCAGC; CPEB4_03_Rv: AAACGCTGCTTCGGCTAATAACGGC. Briefly, oligonucleotides were phosphorylated, annealed and ligated into pSpCas9(BB)-2A-Puro (PX459) V2.0 vector (Addgene, 62988). UACC-62 cells were transfected with 2 µg of each construct using Lipofectamine 3000 (Invitrogen) following manufacturer's instructions. After 24 hr, cells were selected with puromycin (1µg/mL) for 2 days and gene silencing efficiency was determined by immunoblotting. This strategy identified sgCPEB(1) and (3) with depleting activities.

SA-β-galactosidase assay

β-galactosidase staining at acidic pH was performed as previously described[4]. Briefly, 6 days after infection with lentiviral vectors expressing control or CPEB4 shRNAs, melanoma cells were washed twice with phosphate-buffered saline (PBS; pH 7.2), fixed with 0.5% glutaraldehyde in PBS and washed in PBS supplemented with 1 mM $MgCl_2$. Cells were stained at 37°C in X-Gal solution (1 mg/ml X-Gal (Promega), 0.12 mM $K_3Fe[CN]_6$, 0.12 mM $K_4Fe[CN]_6$, 1 mM $MgCl_2$ in PBS at pH 6.0). The staining was performed for 4–6 h to minimize the background signal. Experiments were repeated at least twice in triplicates.

Tissue Immunohistochemistry and immunofluorescence

For CPEB4 detection in benign vs malignant melanocytic lesions by immunohistochemistry, a total of 56 paraffin embedded tissue samples including common melanocytic dermal nevi (N=21), primary vertical growth phase melanoma (N=10), skin (N=14) and lymph node (N=11) melanoma metastases were stained with mouse monoclonal antibody against CPEB4 diluted 1:2000 (clone ERE93C, generated by Monoclonal Antibodies Unit from CNIO) and were analyzed on whole tissue-sections. Samples were processed using BondTM Automated System (Leica Microsystems) by Monoclonal Antibodies Unit from CNIO. After automated dewaxing and rehydration of the paraffin embedded sections, heat-induced antigen retrieval was performed using Bond Epitope Retrieval Solution 2 (Leica Biosystems) and immunodetection was performed with BondTM Polymer Refine Detection (Leica Microsystems) following the manufacturer's instructions. CPEB4 protein expression was scored blinded according to staining intensity and total positive area by two independent dermatologists. The score system used for staining intensity was: 0 (no detectable), 1 (weak), 2 (intermediate) or 3 (high intensity). Similar analyses were performed in tissue microarrays (TMAs) for comparative evaluation of CPEB4 across tumor types (melanoma (N=25), bladder (N=2), breast (N=6), colorectal (N=6), endometrium (N=5), genital (N=3), linfoma (N=6), liver (N=3), lung (N=5), osteosarcoma (N=3), ovary (N=13), pancreas (N=8), thyroid (N=2), non-melanoma skin (N=15) and soft-tissue (N=6) cancers; a total of N=108 specimens).

Additional stainings were performed in paraffin-embedded human skin metastasis or mice xenografts with antibodies against CPEB4 (ERE93C; dilution1:50), RAB27A (HPA001333, Sigma; dilution1:50), MITF (Ab-1, Clone C5, Thermo Scientific; dilution1:400), α-Tubulin (mouse; clone DM1A, Sigma; dilution1:500) and phospho-Histone 3 (rabbit; 06-570, Millipore; dilution1:500). Antigen retrieval was performed using 10 mmol/L sodium citrate buffer at pH 6. Digital images of IHC-stained sections were obtained at 40x magnification (0.12µm/ pixel) using a whole slide

scanner (Mirax scan, Zeiss) fitted with a 40x/0.95 Plan Apochromat objective lens (Zeiss). For immunohistochemical detection, nuclei were counterstained with hematoxilin.

For fluorescence-based analyses, secondary antibodies used were anti-mouse Alexa Fluor 555 and anti-rabbit Alexa Fluor 488 (Life Technologies; dilution 1:400) and DNA was counterstained with DAPI. Negative controls were obtained by omitting the primary antibody. Image mosaics were acquired at 20xHCX PL APO 0.7 N.A. dry objective using a confocal TCS-SP5 (AOBS-UV) confocal microscope. For high-throughput confocal analyses of double IF stainings of CPEB4 and RAB27A in whole-tissue sections, image acquisition was performed using "matrix screening remote control" (MSRC)[5], a new tool for intelligent screening, developed at the CNIO, which improves the quality and speed of image acquisition. In brief, the MSRC tool manages a first fast scan with low-resolution settings to generate one image per slide. This first image is subsequently analyzed by the MSRC software to localize and extract the coordinates of the regions of interest (i.e. tissue samples within the slide). With this spatial information, the MSRC application interacts with the microscope and loads high-resolution settings to scan automatically the areas of interest. After image acquisition, analysis was performed by Definiens XD software, first identifying single cells within every tissue and, then, measuring the fluorescence intensities of green (RAB27A) and red (CPEB4) staining per cell. Similarly, RAB27A and MITF relative expression per tumor was calculated as the product of total positive area and mean intensity of the staining from positive areas.

Supplementary references

1. Ramaswamy, S. *et al.* Multiclass cancer diagnosis using tumor gene expression signatures. *Proc Natl Acad Sci U S A* **98**, 15149-15154 (2001).
2. Wagner, K.W. *et al.* Death-receptor O-glycosylation controls tumor-cell sensitivity to the proapoptotic ligand Apo2L/TRAIL. *Nat Med* **13**, 1070-1077 (2007).
3. Ortiz-Zapater, E. *et al.* Key contribution of CPEB4-mediated translational control to cancer progression. *Nat Med* **18**, 83-90 (2012).
4. Denoyelle, C. *et al.* Anti-oncogenic role of the endoplasmic reticulum differentially activated by mutations in the MAPK pathway. *Nat Cell Biol* **8**, 1053-1063 (2006).
5. Carro, A., Perez-Martinez, M., Soriano, J., Pisano, D.G. & Megias, D. iMSRC: converting a standard automated microscope into an intelligent screening platform. *Sci Rep* **5**, 10502 (2015).
